# A Coalescing Filter for Liquid–Liquid Separation and Multistage Extraction in Continuous-Flow Chemistry

**DOI:** 10.1021/acs.oprd.4c00012

**Published:** 2024-05-06

**Authors:** James Daglish, A. John Blacker, Gregory de Boer, Stephen J. Russell, Muhammad Tausif, David R. J. Hose, Anna R. Parsons, Alex Crampton, Nikil Kapur

**Affiliations:** † School of Mechanical Engineering, University of Leeds, Leeds LS2 9JT, United Kingdom; ‡ School of Chemistry, University of Leeds, Leeds LS2 9JT, United Kingdom; § Chemical Development, Pharmaceutical Technology and Development, Operations, AstraZeneca, Macclesfield SK10 2NA, United Kingdom; ¶ School of Design, University of Leeds, Leeds LS2 9JT, United Kingdom

**Keywords:** liquid−liquid separation, extraction, coalescing media, multistage, counter-current, organic

## Abstract

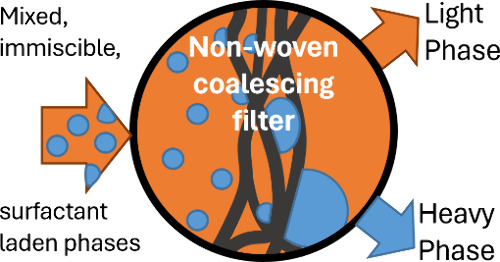

Presented here is the design and performance of a coalescing liquid–liquid filter, based on low-cost and readily available meltblown nonwoven substrates for separation of immiscible phases. The performance of the coalescer was determined across three broad classes of fluid mixtures: (i) immiscible organic/aqueous systems, (ii) a surfactant laden organic/aqueous system with modification of the type of emulsion and interfacial surface tension through the addition of sodium chloride, and (iii) a water–acetone/toluene system. The first two classes demonstrated good performance of the equipment in effecting separation, including the separation of a complex emulsion system for which a membrane separator, operating through transport of a preferentially wetting fluid through the membrane, failed entirely. The third system was used to demonstrate the performance of the separator within a multistage liquid–liquid counterflow extraction system. The performance, robust nature, and scalability of coalescing filters should mean that this approach is routinely considered for liquid–liquid separations and extractions within the fine chemical and pharmaceutical industry.

## Introduction

The economic, environmental, and safety benefits of continuous flow operations in the fine chemical industries have been widely exemplified.^1−4^ Single step reactions performed in flow have resulted in increased yields and purity compared to their batch counterparts. The increased surface-to-volume ratio in continuous flow reactors allow for better mass and energy transfer, smaller reacting volumes that together with the closed nature of the system permits higher temperatures and pressures to be explored resulting in more flexible and diverse reaction systems.^2^ The development of in-line purification technology has expanded the scope of flow chemistry by allowing multiple reaction steps to be performed sequentially in flow.^5^ This has increased the potential applications of continuous processes in the fine chemical industries as more complex molecules with challenging work-ups can be realized via telescoped, multistep continuous flow synthesis.^1,3,6,7^ Furthermore, the development of lab-scale flow technology has enabled the exploration and optimization of new and existing syntheses routes via automated technology and in-line analysis.^8−10^

One purification step that has attracted significant attention in the flow chemistry community is the extraction of products or impurities based on their partitioning between immiscible liquids.^5,11,12^ Liquid–liquid extraction brings benefits including: (i) operation at low temperatures and pressures, of importance when heat sensitive materials require processing;^13^ (ii) high selectivity when combining multiple stages, ideal when components are present in small quantities and require recovery or removal;^14^ and (iii) the ease of use of green and renewable solvents such as those derived from biomaterials which are generally less volatile than crude oil based solvents which are therefore separated more readily by differences in their solubilities rather than by distillation.^15,16^

Several laboratory scale devices have been designed to separate immiscible liquids in order to facilitate liquid–liquid extraction processes. Various phase separation devices have been extensively reviewed elsewhere, particularly those operating on the microscale.^5,17−19^ The two most commonly encountered separation methods are gravity-force driven^20−22^ and surface-force driven.^23−28^ Gravity driven devices rely on density differences between the organic and aqueous phases. For continuous flow operations it is desirable to implement some form of “level sensing” so that the interface between the two phases is kept at a specific height within the device (Table 1). This ensures a given phase exits through the correct outlet. Gravity driven devices are often simple in design and can operate at a range of phase ratios. The range of operational flow rates depend on the separation rate of the two phases and can require a larger footprint^20−22^ than surface force driven devices, which can preclude it from laboratory utilization where reagents are in small supply.

**Table 1:** Gravity Force Driven Separation Devices for Lab-Scale Separations

device	volume (mL)	level control
High-efficiency extraction device (HEED)^20^	3.8	manual pressure adjustment
Computer-vision prototype^22^	2.5	dynamic pump control based on camera level sensing
impedance probe separator^21^	100	dynamic pump control based on impedance probe measurement

At the microscale, surface forces dominate over gravity forces and are therefore suited to utilization in separation of droplets. The underlying principle with surface-force driven separations is that part of the separator will be preferentially wetted by one of the liquids, whether this be a surface, a porous capillary tube or a membrane^23−28^ The differential pressure across the two outlets of these devices must be closely controlled to ensure proper separation and limit breakthrough or retention of the organic into the aqueous phase or vice versa. To overcome this limitation, different methods of controlling the pressure difference have been implemented (Table 2). Surface force driven separation devices work best when interfacial tensions are large and specific flow rates (flow per unit area of membrane area) are small. Operation at phase ratios far from 1 can cause failure of the mechanism, due to the low pressure drop of the flows of the minor phase.^29^ Scale-up can be challenging as surface area to volume ratios need to remain large. Downstream pressure fluctuations and particulates that block small pores or flow channels can cause problems for these separators.^30^ That said, surface force driven separators have small internal volumes and have proven simple to integrate under certain conditions at the laboratory scale. They can separate liquids with similar densities and in some cases break emulsions, meaning residence times are small and what are considered challenging or time-consuming separations in batch can often be dramatically improved via surface force driven separations.^12^

**Table 2:** Surface Tension/Capillary Force Driven Separation Devices for Lab-Scale Separations

device	volume (mL)	outlet control
Asia FFLEX^23^	0.1	manual or automated cross membrane pressure control
Zaiput separator^31^	0.5	automated pressure control via integrated diaphragm
plate separator^25^	<0.01	manual control
steel sieve separator^26^	0.3	manual set pump and pressure-control valve
porous capillary separator^27^	<0.2	optical transmittance sensor controls a needle valve on one outlet—controlling the outlet pressure difference
porous tube based separator^28^	10 nom.	manual cross membrane pressure control

**Figure 1:**
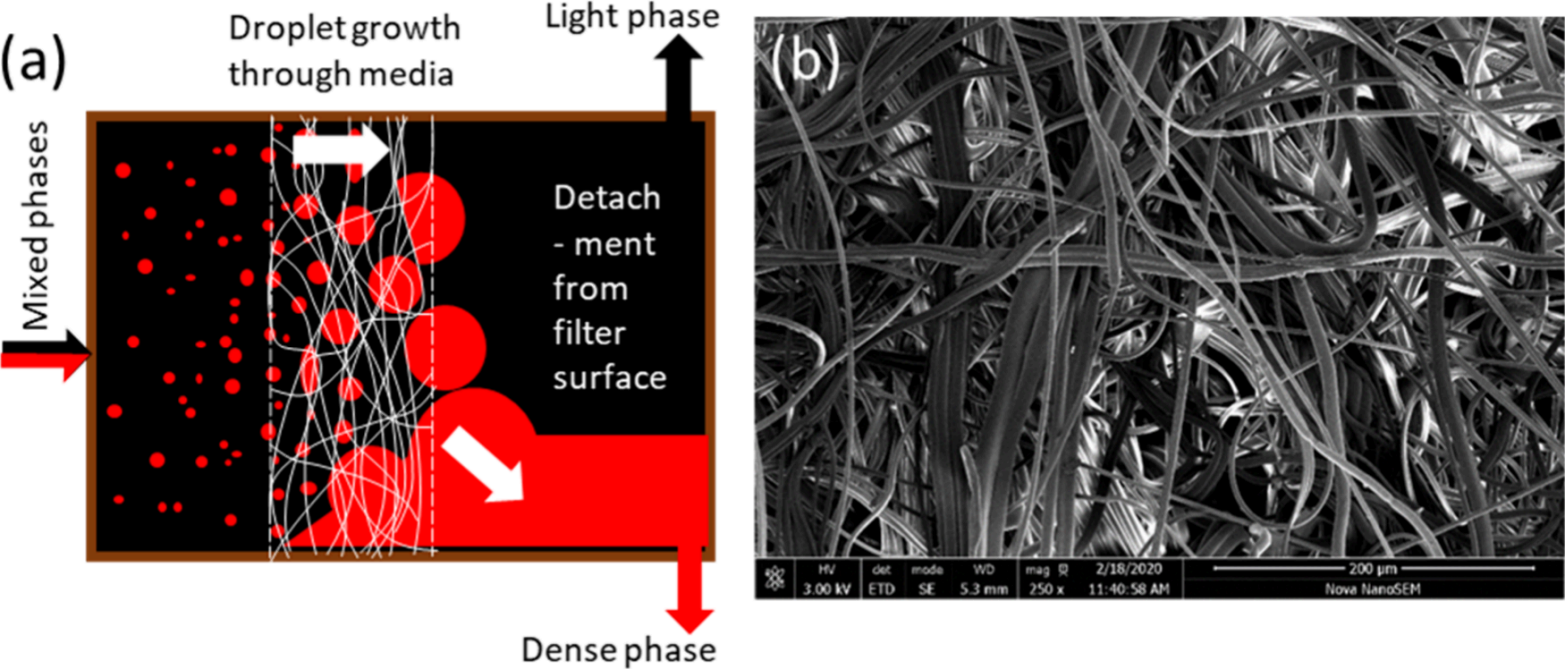
(a) Schematic showing coalescence of droplets on fibers of media, and separation of suspended phase from the continuous phase downstream of the filter. (b) SEM image of meltblown PBT filter media as used in this work (200 μm scale for reference).

One approach that has received little attention within the fine chemical and pharmaceutical industries is coalescing filtration (Figure 1). Coalescing filters make use of both surface forces and gravity forces to separate immiscible liquids. Coalescing filters are depth filters, where the whole of the flow passes through the media, as opposed to surface filters, where one phase is retained on one side of the media. They operate via the following five steps: (i) contact between the dispersed phase droplets and filter fibers; (ii) attachment of small droplets to individual fibers through the depth of the filter; (iii) coalescence of droplets attached to the filter surface; (iv) transport of the enlarged droplets through the filter media; and (v) detachment from the filter surface and removal from the fluid stream via gravity or surface filtration.^32,33^Figure 1a shows a typical separation of a dispersed phase from a continuous phase through a coalescing filter media. Coalescing filters are frequently used in the automotive and aviation industries to remove suspended water from fuel^34^ as well as acting as a guard for solid capture and to separate water from crude mixtures in the petroleum industry.^35,36^

Many different materials have been employed to act as coalescing filters, including thermoplastics such as polyurethane, polypropylene, and polybutylene terephthalate (PBT), glass fibers, paper^33,34,37−41^ and steel meshes.^42^ Each of these materials is used in the form of a nonwoven fabric. Nonwovens consist of fibers with varied orientation that are mechanically, thermally, or chemically bonded. They are distinct from woven or knitted fabrics which are characterized by a repeating structure.^43^ Most polymer based nonwovens used for coalescing filtration are formed via melt-spinning processes and have high porosities and permeabilities, with a median pore size on the order of 10 μm and a highly tortuous fluid path through the media depth.^44^Figure 1b is an SEM image of a typical meltblown PBT nonwoven fabric, which we selected for this study as it is low cost, relatively chemically resistant, and finds wide application including in the medical field^45^ where production of materials must adhere to good manufacturing practices (GMP).

Studies have shown how different filter media and the properties of the liquid–liquid systems can affect the separation performance of coalescing filters. A principle component analysis of a polyurethane nonwoven filter media^35^ showed a strong correlation between an upper critical face velocity where the filter did not perform effectively and properties of bed permeability, oil viscosity, interfacial tension, emulsivity, and dielectric constant. Surface energy and the pore size have both been highlighted as controlling factors,^33^ with enhanced coalescence at reduced pore size and at surfaces with preferential wetting toward the disperse phase. Similarly, the wettability of the filter media can influence the pressure drop across the filter with more hydrophilic membranes giving greater separation but with an increased pressure drop across the media attributed to water droplets being retained within the filter media.^34^ The microstructural design of the porous media has also been shown to influence the efficiency of separation with a decrease in pressure drop and increase in efficiency through introducing a pore size gradient^36^ or a wettability gradient^41^ when compared to homogeneous media.

A coalescing filter (a term defined herein to indicate a coalescing depth filter based on the use of nonwoven fibers) is therefore a potentially very effective method for separations during multistep synthesis that is suited to both process design and scale-up. To that end we have developed a laboratory-scale coalescing filter to separate immiscible liquids and a conductivity measurement based PID controller to control the relative outlet flow rates by varying either the position of a needle valve or the flow rate of a diaphragm pump. The device has been integrated into a typical flow chemistry lab system utilizing miniature continuous stirred tank reactors which incorporate a magnetically coupled stir-bar (fReactor CSTRs, Asynt Ltd.)^46^ to create the initial emulsion. We demonstrate performance across a range of organic-aqueous systems, including a particularly challenging surfactant system, and compare results against those of a membrane separator. Finally, we demonstrate how the units can be configured for a counter-flow multistage extraction.

## Coalescing Filter Design

### Mechanical Design

**Figure 2:**
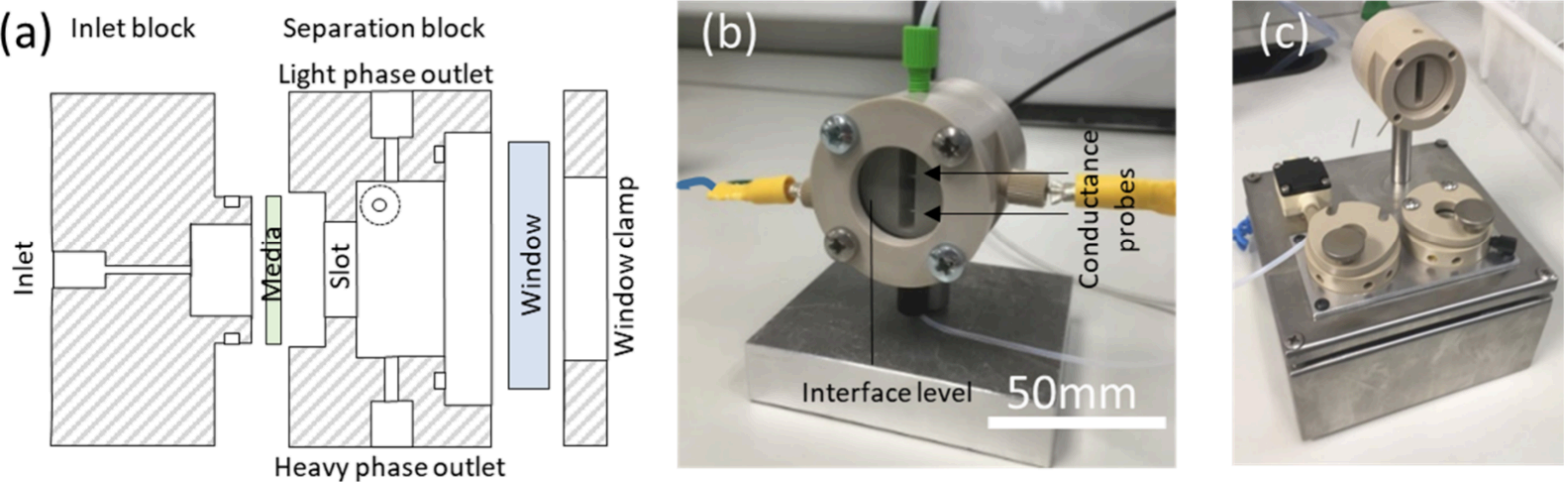
(a) Sectional view of the coalescing filter housing. Photograph of (b) the assembled laboratory scale coalescing filter and (c) assembled coalescing filter, CSTR fReactor mixers and pump.

Figure 2 shows the design of the coalescing filter. With reference to Figure 2a, the stub of the inlet block inserts into the separation block, with the filter media located between the faces of these blocks. The inlet is located centrally on the inlet block. Within the separation block, is a slot (5 mm wide, 15 mm high) which defines the working area of the filter. An initial design used a larger 15 mm diameter working area, but the high separation efficiency of the filter meant breakthrough (and failure of the separation) was difficult to achieve as high flow rates were required and differentiation of performance was not easy to show. The move to a smaller working area also reduced the internal volume to just 2 mL. Downstream of this is the separated zone (25 mm diameter) where the separated heavy and light phases flow out of the coalescing filter through two outlets located at 6 o’clock and 12 o’clock positions, respectively. A glass window locates on the face of the main body, to allow visualization of the separated fluid phases. PTFE coated Viton o-rings seal the fluid path, with one located within a groove on the outside diameter of the inlet block, and a second between the borosilicate glass window and the separation block. All parts are machined from PEEK, with all fluid and electrode ports accepting standard 1/4–28 UNF low pressure fittings (Figure 2b). The coalescing media was a cut disc of melt- blown PBT (selected for its general chemical resistance and high melting point of 220 °C) either used directly as received from the manufacturer or treated to render it more hydrophilic. Characteristics of the media are given in the materials section.

### Level Control System

**Figure 3:**
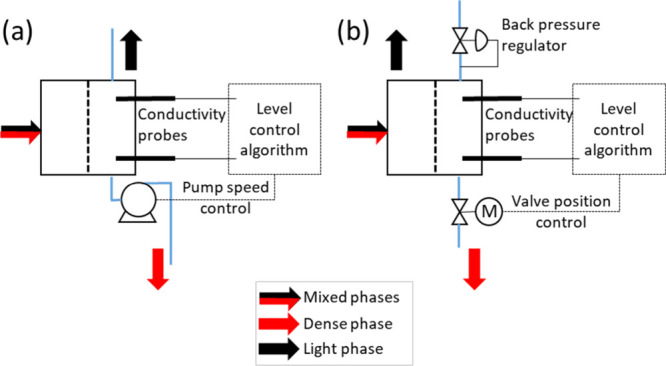
Schematics of (a) the pump actuated control system, (b) the valve actuated control system.

The relative outlet flow rates of the aqueous and organic phases are controlled to ensure no crossover in the separated streams. To do this, active control is used to set the interface level downstream of the valve, with conductance used to infer the height of the interface between the two fluids. Two electrodes (stainless steel) are inserted into the separated zone downstream of the media (Figure 2b), to allow the interface height to be inferred through a measure of conductivity, which proved effective for all the aqueous–organic systems studied here with the aqueous phase having significantly higher conductivity than the organic phase. The output of the algorithm is used to control either (i) a pump rate (Figure 3a) or (ii) a valve position using a servo driven needle valve (Figure 3b). The former allows for multistage extractions and the latter is somewhat simpler in design, although performance is otherwise similar for single stage systems. For consistency, all studies assessing separation efficiencies described later were carried using the pump actuated control system (Figure 3a).

The pump system used a KNF FEM 1.02 diaphragm pump (Supporting Information, SI, Figure S1), which contained wetted materials of PTFE (diaphragm), PVDF (pump body), and FFKM (gaskets and o-rings), which generally show good solvent compatibility. Implementing the pump system allows for multistage extractions, as will be demonstrated later. The valve system consists of a PEEK micrometering needle valve on the heavy phase outlet fitted with a servo (SI Figure S2). A back-pressure regulator (BPR) was placed on the light phase outlet to ensure that when the valve is fully open, fluid will only flow out of the heavy phase outlet, and when the valve is closed the fluid will only flow out of the light phase outlet. In either case, an electrical conductivity circuit (Atlas Scientific) was used, with the control algorithm coded onto an embedded processor (Arduino Mega 2560). Further information, including the interface between the processor and the pump or servo can be found in SI Section 2.

### Control Algorithm

Given its extensive track-record in offering flexible control, a PID controller was implemented (using the PID Arduino library developed by Beauregard, 2011^47^) to maintain the interface level. A brief synopsis is now given, with a more detailed explanation of the method given in SI Section 2. Two elements are required to establishing the overall control scheme: (i) defining a suitable conductivity set-point, since the level is only inferred from this measurement; (ii) identifying suitable values for the *K*_P_, *K*_I_, and *K*_D_ constants (corresponding to the proportional, integral, and derivative components, respectively) of the controller.

#### i Defining the Operating Set Point

Preliminary tests, using a water–toluene system, showed that stable flows were achieved when the conductivity set point was set at 90% of the actual conductivity of the aqueous phase (maximum measurable conductivity of the solution). This indicates the interface level is between the two electrodes within the body of the filter (Figure 2b).

To establish this set point automatically, an initialization procedure was developed where the pump was stopped, or the valve fully closed, allowing the conductivity probe to become fully submersed in the aqueous phase giving a readout of the maximum conductivity of the aqueous solution. The new conductance set point, was then set at 90% of this value.

#### ii Identifying the PID Constants

**Figure 4:**
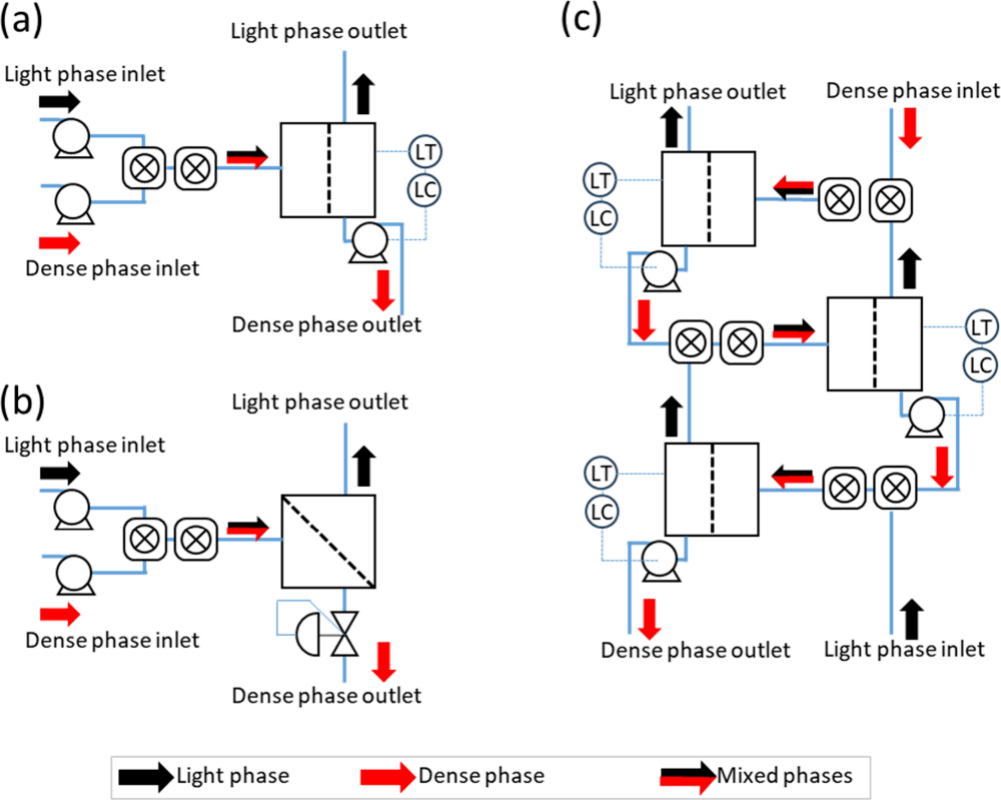
Flow schematics for single stage separations using (a) coalescing separator level controlled with pump and (b) membrane separator with (integral) pressure control. (c) 3-Stage counter-current multistage separator using coalescing media.

with the set point now established, the PID constants *K*_P_, *K*_I_, and *K*_D_ were determined using the flow arrangements in Figure 4a,b, using the water–toluene system, with a flow rate of 4 mL/min. A screen was used to establish the lowest standard deviation in the conductivity, which corresponds to the most uniform value of valve position or pump speed. The resulting values of the PID constants were found to be *K*_P_ = 1, *K*_I_ = 0.05, and *K*_D_ = 0.5. These parameters were found to give effective control of the interface layer for all subsequent tests.

## Materials

### Filter Media

The coalescing filter material used in this study was a hydrophobic meltblown PBT fabric provided by Mogul Co. The material was characterized in terms of area density (40.4 ± 0.8 g/m^2^), thickness (0.39 ± 0.01 mm), intrinsic permeability (5.7 × 10^–11^ ± 8.0 × 10^–12^ m^2^), porosity (92%), and a mean pore size (20.1 μm) with details of the measurement techniques given in SI section 4. The hydrophobic nature of the PBT will give preferential wetting to the organic phase; this is beneficial when the organic forms the dispersed phase, allowing the fibers to retain the droplets until they are larger. When the dispersed phase is aqueous, it may be beneficial to increase the hydrophilicity of the filter, allowing the aqueous droplets to wet the fibers thus giving a correspondingly larger size of the separated droplet. Following Arouni et al. (2019)^34^ and Wang et al. (2014),^48^ hydroxyl-end groups were formed on the polymer using 3 M sodium hydroxide solution in a methanol/water mixture (1:1 by volume) heated to 40 °C for 10 min, before rinsing until neutral. The untreated hydrophobic media (contact angle 133°) and treated hydrophilic media (contact angle <90°) (SI section 4) allowed the influence of surface properties on separation performance experiments to be assessed.

### Liquid Test Systems

Three classes of fluid test systems were adopted for characterization of the coalescing filter. For the single stage system, four pure aqueous/organic mixtures (referred to as the class (i) system) and a more complex surfactant loaded aqueous/organic system (class (ii) system) were used to demonstrate phase separation performance. The latter is particularly relevant within the chemicals industry, as emulsions can form in chemical manufacture due to the interplay of phase composition, mixing regime, acids/base addition, byproducts, additives, catalysts, solvents, and impurities.^49−51^ The undesirable formation of an emulsion cannot always be avoided without reworking a manufacturing method or compromising on yield/purity. Coalescing filters have the potential to separate fine emulsified droplets, which may be stabilized by a surfactant.^33−36,41^ For the multistage extraction, the partitioning of acetone from an aqueous–acetone feed into a toluene stream was used (class (iii) system). The removal of acetone from water has been used multiple times as a standard test system to characterize extraction equipment and processes.^52−54^ The system is inexpensive to use and is well understood thermodynamically, making it simple to model and obtain equilibrium data for single or multiple stages of extraction. This system also gives a volume change between the aqueous and toluene stream so can be used to demonstrate the resilience of the control strategy.

#### Class (i) System

The four pure immiscible aqueous/organic pairs of liquids are summarized in Table 3. The organic phases were chosen to cover a range of interfacial surface tensions (IFTs) and densities. IFTs were measured with the pendant drop technique on a Kruss DSA100. The stability of these emulsions was determined through a shake-test followed by an image-based separation and conductance measurement.^55^ Where the emulsion had a high conductivity reading it was O/W, a low conductivity reading was W/O and an intermediate conductivity measurement suggested a mixed phase emulsion. This data is shown alongside the results of the separations (Table 5).

**Table 3:** Properties of the Pure Test Systems^a^

aqueous phase	organic phase	interfacial surface tension (mN/m)	organic phase density(kg/m^3^)	organic phase viscosity (mPa·s)
water	toluene	36.5	867	0.56
water	ethyl acetate	6.4	902	0.426
water	1-butanol	1.8	810	0.26
water	dichloromethane	28.9	1330	0.413

a The aqueous phase has a density of 1000 kg/m^3^ and viscosity of 1 mPa.s. All values reported at 25 °C.

#### Class (ii) System

For the emulsion system, a toluene–water–surfactant system,^55^ with a 0.01 M aqueous solution of sodium dodecylbenzenesulfonate (SDBS) as the surfactant was adopted, since this system gives a range of emulsion stabilities depending on the level of salinity (NaCl in this case). Four samples (A–D) shown in Table 4 were adopted to cover a range of stabilities with concentration of NaCl varying from 7.8 mM to 470 mM. The hydrophilic lipophilic difference (HLD) theory can be used to interpret the samples^56−58^ as it gives a measure of the separation performance of a two-phase mixture with surfactants. Where HLD is close to zero (sample D), the interfacial surface tension is very low and coalescence of droplets is rapid. As the HLD value moves away from zero, then separation times increase exponentially (samples D → C → B) until values of HLD still further from zero result in an increased interfacial surface tension, giving a reduced separation time (samples B → A). The settling performance of these specific samples have previously been studied more completely in ref (55), where further details of the calculation HLD value is given.

**Table 4:** Summary of Emulsion Separation Results for Surfactant System^a^

sample	NaCl concentration (M)	IFT (mN/m)	HLD value	emulsion type	top phase separation time (min)	bottom phase separation time (min)
A	0.0078	3	–3.38	O/W	>120	41.7
B	0.18	2	–0.91	O/W	>120	>120
C	0.37	<1	–0.23	mixed	>120	79.2
D	0.47	≪1	0.01	mixed	2.33	34.8

a The top and bottom phase separation times, measured from a shake-test, indicate the stability of the system, with longer separation times indicating more stable emulsion systems.

#### Class (iii) System

A 50% w/w solution of acetone in water was used as the aqueous feed (with 250 mg/L of NaCl added to give a high conductivity reading), with 99.8% anhydrous toluene used as the extracting solvent, in line with previous studies.^52−54^

## Experimental Methods

### Coalescing Filter Performance for Single Stage Separations

To determine how well the coalescing filter performed at various flow rates and phase ratios, a series of test runs were conducted using class (i) and (ii) systems identified above, with the performance of the coalescing filter characterized by the percentage volume of each phase that crossed over into the other phase’s outlet. A perfect separation would have 0% aqueous phase in the organic outlet and 0% organic phase in the aqueous outlet. Quantification of the sample was carried out by collecting 12 mL of the outlet stream in a constant cross-section vial (SI sections 5 and 6) before imaging to establish the separation. An identical study was carried out using a commercial membrane separator (the Sep 10 Zaiput^31^), allowing for comparison between coalescing filtration and membrane separation.

The tests were carried out using the flow circuit shown in Figure 4a, with the actual unit shown in Figure 3c. Two Jasco PU HPLC pumps (model 1580 or 1585) were used to transfer the organic and aqueous phases to a first fReactor CSTR^46^ (volume 1.6 mL) with the outlet stream from this passing into a second fReactor CSTR before flowing into the coalescing filter. Both the residence time and the degree of mixing, together with the physicochemical characteristics of the two phases and surfactants, will all influence the droplet size distribution and the stability of the resulting emulsion.^58,59^ We control, but do not explicitly measure this by maintaining a constant mixing speed of 1000 rpm within the two fReactor units. Two units were used, as this provided enough interfacial contact for mass transport between phases to reach an equilibrium (see results section: Counter-current multistage extraction system). An identical feed circuit of HPLC pumps and fReactors was used with the Sep10 Zaiput^31^ membrane separator (Figure 4b).

For the class (i) system, the performance of the coalescing filter was evaluated without filter media, with 10 layers of untreated and with 10 layers of treated filter media. Fresh filter media was used for each test. The membrane separator was tested with both a hydrophobic and hydrophilic membrane (OB-900 and IL-900). In all cases, the results from the best performing membrane were reported. Each liquid–liquid pair was tested at 4, 10, and 16 mL/min (total flow rate) at a phase ratio of 1, and at 10 mL/min (total flow rate) at a phase ratio of 0.25 and 4.

For the class (ii) system, the coalescing filter was tested with 0, 1, 5, and 10 layers of treated media, at a phase ratio of 1 and a total flow rate of 5 mL/min. As before, fresh filter media was used for each test. The membrane separator was tested with this system using both the hydrophobic and hydrophilic membrane (OB-900 and IL-900).

### Coalescing Filter Performance—Counter Current Extraction

To establish performance for multistage separations, tests were carried out using the class (iii) system identified above for 1, 2, and 3 stages, with an example of the 3-stage counter-current circuit shown in Figure 4c. Each stage of separation consisted of two miniature CSTRs to mix the two phases and a 2 mL separator with 10 layers of hydrophobic PBT filter media with an interstage pump connected to its aqueous outlet (Figure 2c), giving a volume of 5.6 mL per stage. The efficiency of extraction was evaluated with phases ratios of 1:1, 2:1, and 3:1 (aqueous to organic), under a fixed inlet flow rate of 3 mL/min (giving 9 measurements in total). The extraction efficiency was evaluated through GC analysis of the toluene stream exiting the reactor, with the peak area of toluene (and its insoluble nature in water^60^) used to establish the overall organic flow rate leaving the extractor, and the peak area of acetone used to calculate the fraction of acetone. Further information including calibration curves can be found in SI section 7.

## Results and Discussion

### Separation of Pure Solvent Systems (Class (i)

The results of separations of the pure solvent systems using both the coalescing separator and the membrane separator are shown in Table 5, alongside the nature of the separation media used. For both cases, (if there was a difference) the result of best performing type of separation media has been presented.

**Table 5:**
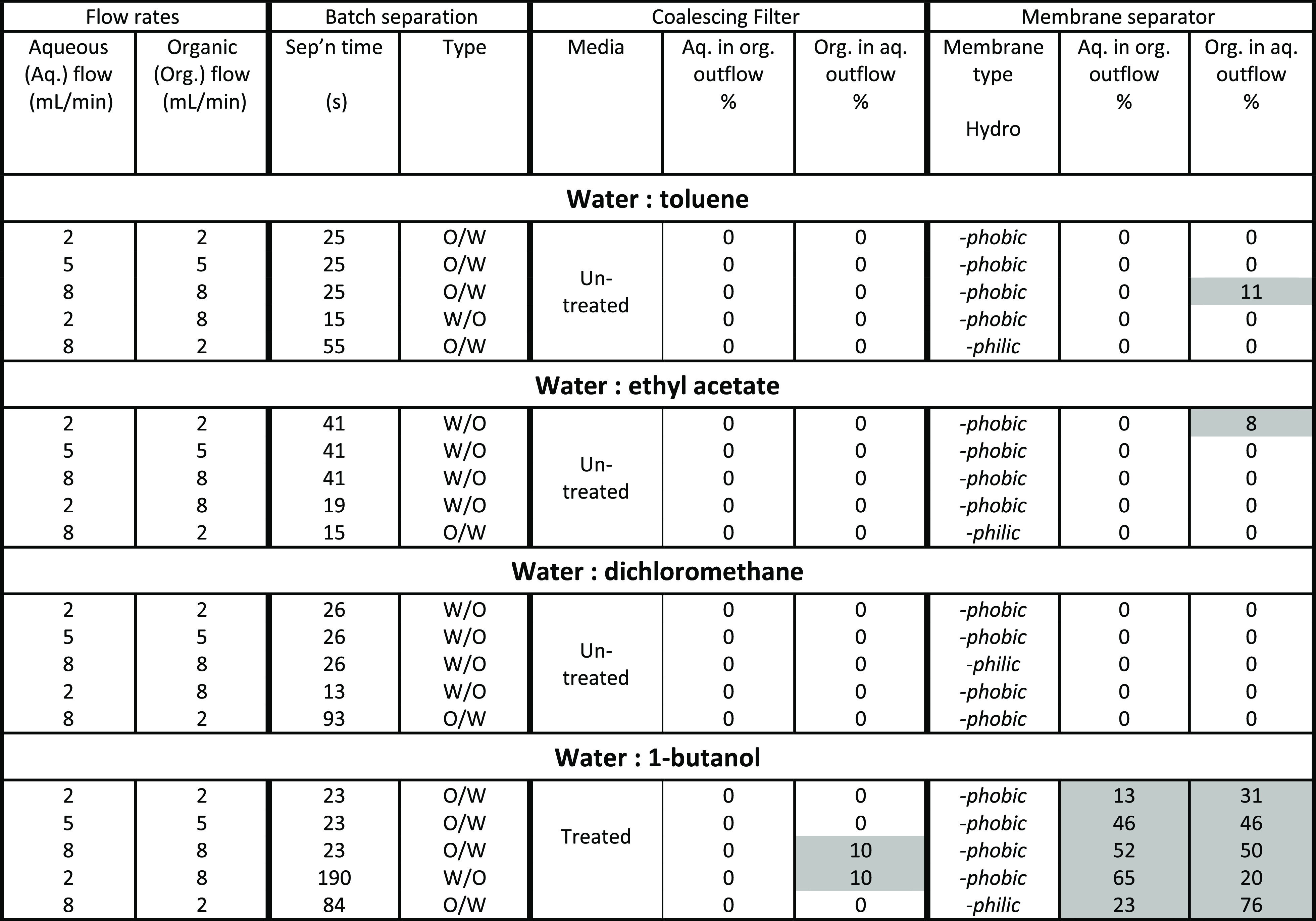
Summary of Coalescing Filter and Membrane Separator Performance^a^^,^^b^

a Data highlighted in grey are non-zero, representing phase crossover.

b Untreated coalescing filter is hydrophobic, treated coalescing media is hydrophilic.

Generally, the coalescing filter performed excellently across all the solvent systems, with perfect separations in all except two conditions, while the membrane separator failed for one of the systems and showed crossover in two others. We make the following observations about the different solvent systems.

Water–toluene has a relatively high IFT and difference in density, and the shake-test separation times are relatively short at all phase ratios. With the coalescing filter, the untreated media was most efficient at effecting the separation and did not depend on the emulsion type. Similar behavior was observed for the water–ethyl acetate system, which has a lower interfacial tension but otherwise similar physical properties. The coalescing filter gave efficient separations over all studied flow conditions. In terms of the membrane separator, imperfect separation happened at the higher flow rates (16 mL/min, 1:1 phase ratio) for the water–toluene system and the lowest flow rates (4 mL/min, 1:1 phase ratio) for the water-ethyl acetate system. Sixteen mL/min is above the recommended flow rate of the membrane separator and so some failure of the device was expected at this flow rate. The failure to separate ethyl acetate at 4 mL/min is harder to explain but may be due to the sensitivity of the pressure control at these lower flow rates. Using a smaller pore membrane may have reduced the crossover in this case.

The use of dichloromethane shows the ability of the coalescing filter to operate when the organic phase has a higher density than water, meaning that the aqueous layer would be upper-most within the coalescing filter. The only change required is to rotate the separator housing by 180°, to ensure one conductivity electrode is permanently within the aqueous layer and both electrodes can be submerged in the aqueous phase. This demonstrates that the coalescence separator is just as effective at separating organic phases that are denser than water as for those (more common cases) when the organic phase has a lower density.

1-Butanol has the lowest interfacial tension with water at 1.8 mN/m and so presents a significant challenge for both the coalescing filter and membrane separator as both rely on the two phases having different wettability. For the coalescing filter, at a phase ratio of 1:1, carry over of the organic phase into the aqueous phase was observed at higher face velocities. From the mechanistic understanding of the coalescing filter, the higher velocities may be stripping droplets from the fibers and re-entraining these into the aqueous stream. However, since complete separation is possible at the lower face velocities, the separation of water–1-butanol is entirely feasible with the coalescing filter, albeit with a slightly larger specific area of media. The membrane separator failed to separate this system, despite a range of membrane types being evaluated and in general the performance of the membrane separator was observed to decreases with increasing flow rate.

**Figure 5:**
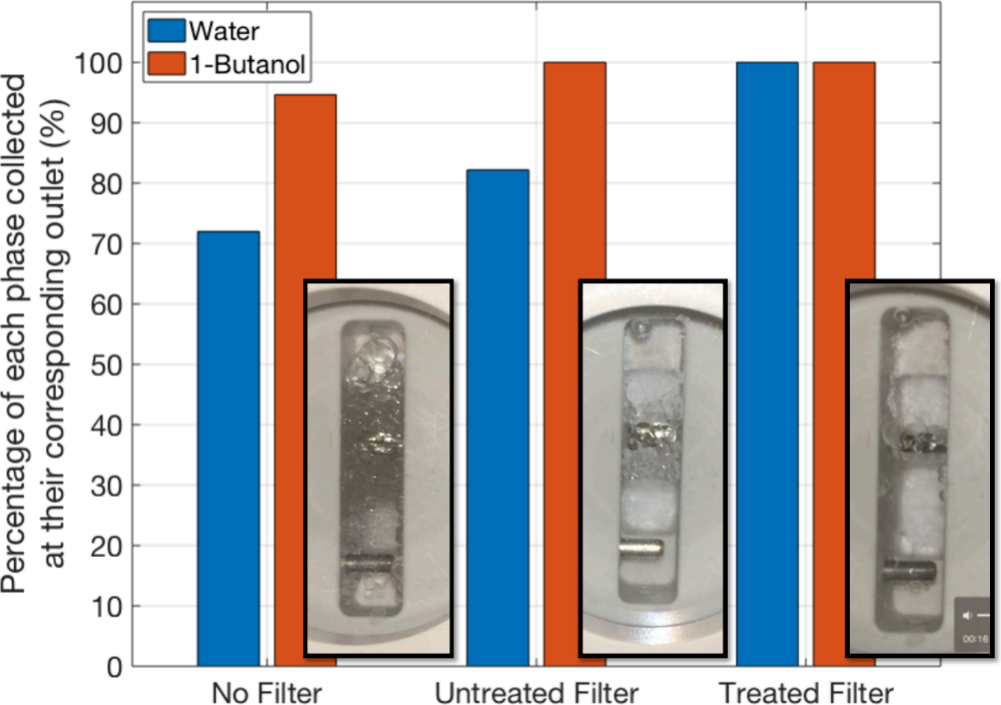
Percentage of water and 1-butanol collected at each outlet of the coalescing filter during operation at 10 mL/min (total) and image of separation on downstream side of media.

For the 1-butanol system, Figure 5 shows images of the downstream side of the media within the coalescing filter for the case of no filter, untreated, and treated filter media (5 mL/min flow per phase). This confirms that gravity separation is not the only mechanism contributing to the separation as both the untreated and treated filter media separate the two phases better than when there is no filter. Increasing the hydrophilicity of the filter increased the amount of water collected in the aqueous outlet from 82% to 100% (perfect separation). The size of the emulsion layer in the coalescing filter can be seen to significantly reduce when the more hydrophilic treated filter media is used in place of the untreated filter media, demonstrating the effectiveness of the coalescing filter media in separating these immiscible liquids.

### Separation of Surfactant System (Class (ii)

**Figure 6:**
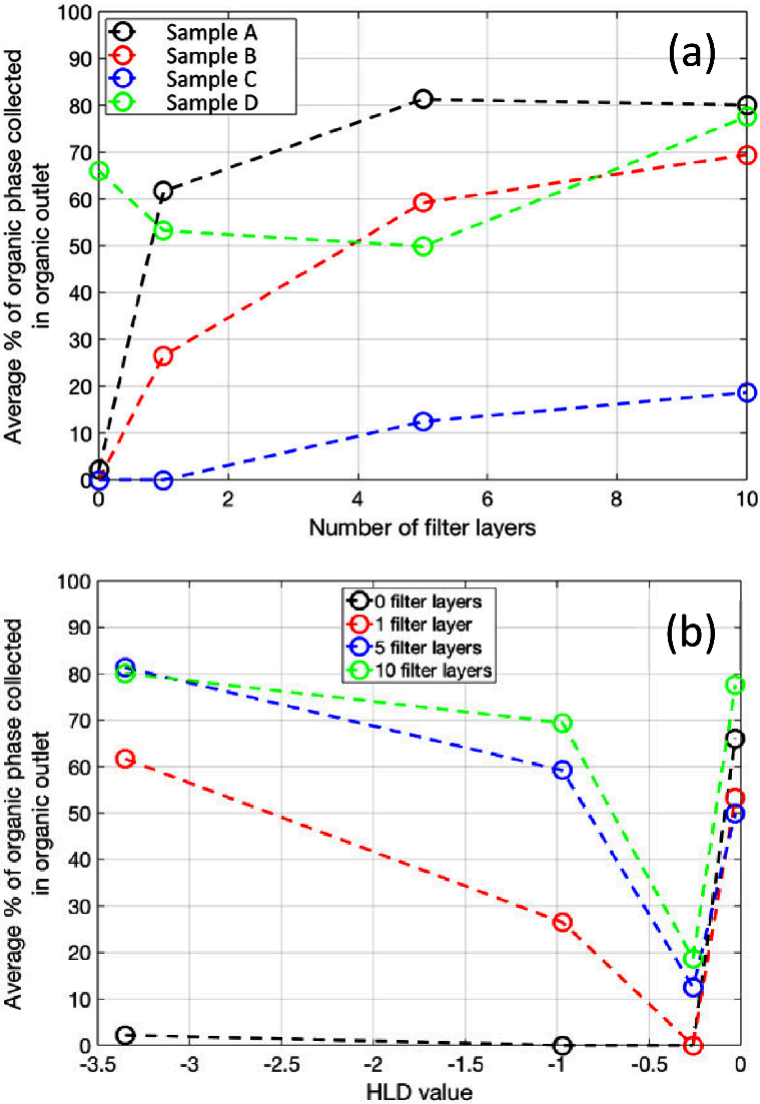
Average percentage of organic phase collected at the organic outlet depending on the (a) number of filter layers and (b) as a function of the HLD values.

**Figure 7:**
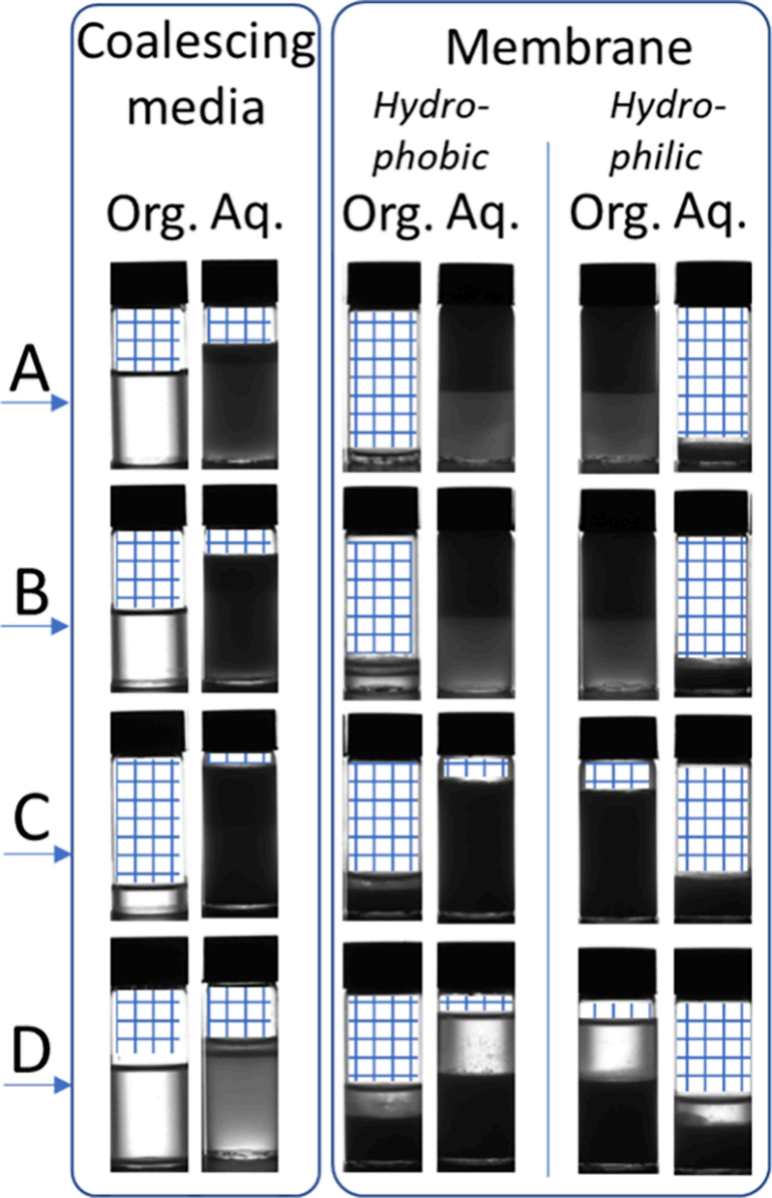
Images of samples collected at the organic and aquous outlet for water–surfactant–toluene mixtures (A–D, Table 4). The blue hatch has been added to indicate where no liquid is present within the vials.

Figure 6 shows the results of the separation efficiency for the coalescence media for the four emulsion systems. Sample A and B, with the most negative HLD values show an improved separation as the number of layers of media is increased (Figure 6a)—a thicker layer gives a longer tortuous flow-path and more opportunity for droplet–fiber interaction. Despite both these systems showing high stability in batch testing (>2 h, Table 4), the coalescing filter is capable of separation of the aqueous and organic phase to a relatively high level. The organic phase recovered from these samples is transparent suggesting no second phase is present—see Figure 7 (samples A and B, coalescing media column).

At HLD values close to zero, the behavior is more complex (Figure 6b). Samples C and D are both mixed emulsion types, so the interface structure between the two phases is complex (e.g., water in solvent in water). Where the HLD value is closest to zero, the interfacial surface tension is lowest (sample D, Table 5) and the barrier of coalescence for a given phase is reduced. Sample C is a stable mixed emulsion system, making for a complex interaction between a given phase of droplets and the fibers of the coalescing media.

The ability of the coalescing filter to separate all samples except C is promising, particularly given the stability of the samples (samples A and B were not observed to separate under gravity over a 2-h testing period—Table 4). This also illustrates the importance of rationally understanding the physicochemical properties of the mixture which provides further opportunity for optimization of the system. In this case by adding more sodium chloride to sample C will give an HLD value closer to zero and allow separation. The separations of these systems have been carried out with little optimization of the coalescing media itself, and this may be a fruitful area to explore.

The performance of the membrane separator is in stark contrast to the coalescing filter. The membrane separator failed to separate the two phases, using either a hydrophilic or hydrophobic membrane. For samples A and B the entire outflow was from one outlet only, and for samples C and D there was some outflow from both channels but, at least qualitatively, there seemed to be little difference in the ratio of organic to aqueous fractions in each outlet (Figure 7). This is unsurprising, as the interaction between surfactants and surface will cause the membrane to foul^61^ offering no selectivity in transport of one phase over the other through the membrane.

### Counter-Current Multistage Extraction System (Class (iii)

**Figure 8:**
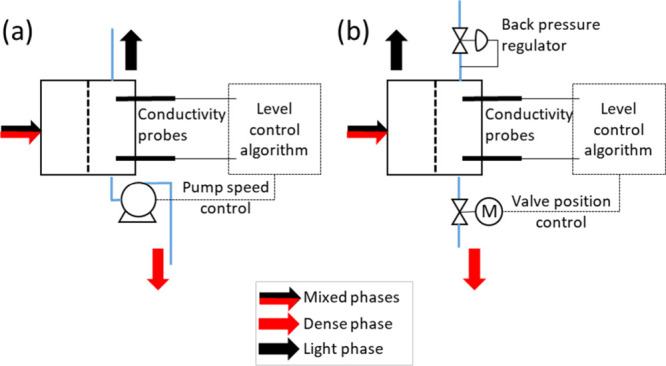
Percentage of acetone extracted from the aqueous phase, depending on phase ratio and number of extraction stages. The *x*-axis label S#_1_PR#_2_ shows the number of stages (#_1_) and the phase ratio of aqueous to organic phase (#_2_). The extraction percentage is compared to the percentage found in batch as well as by the Aspen thermodynamic model. Batch data for single step extraction is shown, together with predicted results for sequential, multistep batch extractions.

The results from the 9 experimental runs are shown in Figure 8. The *x*-axis label S#_1_PR#_2_ shows the number of stages (#_1_) and the phase ratio of aqueous to organic phase (#_2_). As the number of stages is increased for a given phase ratio (S1 → S2 → S3), the recovery of acetone is observed to increase. One, two, and three stages operated at a phase ratio of one show recovery of acetone of 62%, 91%, and 96%, respectively. As the phase ratio of aqueous to organic is increased for a given number of stages (PR1 → PR2 → PR3), then the recovery of acetone becomes more challenging as there is a reduced driving force to partition into the toluene. Generally, there is good agreement between experimental values and those from the Aspen thermodynamic model (SI section 8).

Single stage batch extractions, where the aqueous and organic phase were mixed and allowed to separate under gravity, showed good agreement with the single-stage counter-current extraction which indicates that the mixing within the CSTRs (Figure 4) is such that equilibrium is reached. The largest difference between the batch experiments and the single stage counter-current extraction system was at a phase ratio of 1, with a 6% difference—attributed to error on the pump flow rate, or a slight modification in separation efficiency due to the salting out effect.^62^

Finally, a brief analytical study was carried out to illustrate the differences between continuous counter-current separation, using the coalescing separators and a multistep batch extraction, as commonly encountered in process industries. Each step of the batch extraction consists of (a) mixing the aqueous phase and organic phase; (b) allowing separation and draining the aqueous phase before remixing the aqueous phase with fresh organic phase in the next step. Here, a phase ratio of 1 was studied (PR1), across 1, 2, and 3 stages. For the batch extractions, the organic phase was divided equally between each of the extraction steps that were to be carried out, to give an equivalent basis for comparison with the counter-current separation. The previously calculated tertiary phase diagram was used to calculate the batch compositions at each step (SI section 9). Results are shown in Figure 8. In line with previous studies,^63^ a multistep batch extraction is always more efficient than a single step, despite using the same overall volume of organic phase, and the counter-flow separation over *m* stages is more efficient than *m* steps of batch extraction, in this case by ∼10% (2 or 3 stages).

The performance of the control algorithm, coded into each individual pump and separator unit, was found to be consistently capable of establishing suitable flow rates without crossover. It was able to appropriately control the interstage flow rate which changes due to the volume effect of acetone partitioning from the aqueous stream (reducing the aqueous flow rate) into the toluene stream (increasing the organic flow rate). The largest change in organic phase flow rate was found after 3 stages at a phase ratio of 3 where the flow rate increased by a factor of 2.6 (0.75 mL/min to 1.94 mL/min) (SI section 10). This demonstrates the general robustness of the control system for both single stage and multistage extractions, and the ability to add additional stages to improve the overall separation efficiency.

## Conclusions

The design of a robust small-scale laboratory-based separator and associated control system using conductivity and a PID algorithm has allowed the study of coalescence-based separations for a range of organic and aqueous systems. This includes separation in the presence of surfactants, across a range of interfacial surface tensions and density differences. In particular, the presence of surfactants can challenge the effectiveness of separators, as the surfactants can adsorb to surfaces changing the wetting characteristics (“fouling”^61^). The coalescing filter showed promising results for separating a complex surfactant-aqueous-solvent mixture, whereas a membrane separator failed entirely. Understanding the physicochemical properties of the emulsion and knowledge of how these can be modified (e.g., by adjusting the salinity), together with further optimization of the properties of the nonwoven material will bring further performance gains.

The coalescence separator was also used within a multistage counter-current liquid–liquid extraction circuit and proved an efficient way to recover a solvent phase from an aqueous stream, with increasing number of stages giving a higher overall recovery, in line with predictions. An equivalent number of sequential batch extractions gives a lower recovery; the water/acetone–toluene system used here separates easily under the influence of gravity alone after mixing, but where systems have long separation times then a multistep batch extraction becomes time-consuming. The continuous counter-flow extraction using coalescing media to drive the separation requires just one pass through the flow system and gives an improved recovery.

For porous media flows, the face velocity provides the relevant scale-up parameter and will allow tests using the small-scale experimental equipment described here to inform scaleup. The technical requirements for nonwoven coalescing filter media compared to membrane separators is low and consequently is both cost-effective and performance enhancing. Typical face velocities through the coalescence separator were 2 mm/s, giving a specific throughput of 120 L/(min m^2^). Operating closer to the critical face velocity for a given system could increase this further. Subsequent use of the coalescing filter in the selective extraction of one amine from a binary mixture has shown long stability of the media over many weeks of periodic operation^64^ with further work required to confirm performance at larger scales.

This work demonstrates the potential of coalescing filtration as a valuable tool in liquid–liquid separations and in multistage extractions within the fine chemical and pharmaceutical industry. Generally, we find there is a lack of understanding of the capability of coalescing filtration, for example Chemical Engineering Design^65^ states “*Coalescing Filters are suitable for separating small quantities of dispersed liquids from larger throughputs*” whereas this work demonstrates that the technical capability of coalescing filters, both for separation and by incorporation into multistage liquid–liquid extraction, is far greater than perhaps more generally acknowledged.

## Supporting Information Available

Technical detail of the equipment and control system; measurement protocols for filter media characterization; images and raw data of the separation performance; measurement protocols for counter-current extraction of acetone and model development for counter-current extraction and sequential batch; and volume data from counter-current operation (PDF)
