# Supplementary material for: A Coalescing Filter for Liquid–Liquid Separation and Multistage Extraction in Continuous-Flow Chemistry
Source: Org Process Res Dev. 2024 May 6;28(5):1979–89. doi: 10.1021/acs.oprd.4c00012 (PMC11110050; doi:10.1021/acs.oprd.4c00012)
Supplement: Supplementary file 1 — op4c00012_si_001.pdf [file op4c00012_si_001.pdf]

ESI to A coalescing filter for liquid-liquid separation and multistage extraction in continuous-flow chemistry

James Daglish[1], A. John Blacker[2], Gregory de Boer[1], Stephen J. Russell[4], Muhammad Tausif[4], David R. J. Hose[3], Anna R. Parsons[3], Alex Crampton[3], Nikil Kapur[1*].
[1]School of Mechanical Engineering, University of Leeds, LS2 9JT,
[2]School of Chemistry, University of Leeds, LS2 9JT.
[3]Chemical Development, Pharmaceutical Technology and Development, Operations, AstraZeneca, Macclesfield, SK10 2NA, UK.
[4]School of Design, University of Leeds, Leeds LS2 9JT, United Kingdom

*Corresponding author: n.kapur@leeds.ac.uk

ESI Section 1 – VALVE AND PUMP SYSTEM	***S2***
ESI Section 2 – ELECTRONIC CIRCUITS AND INTEFACE WITH FLUID CIRCUIT<br>**S2.1: Valve system**<br>***S2.2 Pump system***	***S4***
ESI Section 3 – CONTROL SYSTEM DEVELOPMENT<br>***S3.1 Development of PID Control System***<br>***S3.2 Standard Deviation of Conductivity and Valve Position***	***S6***
ESI Section 4 – MEASUREMENT PROTOCOL FOR FILTER MEDIA CHARACTERISTICS	***S9***
ESI Section 5 – SEPARATION DYNAMICS OF CLASS I SYSTEM (PURE ORGANIC / AQUEOUS MIXTURE)	***S10***
ESI Section 6 – SEPARATION DYNAMICS OF CLASS II SYSTEM (SURFACTANT / ORGANIC / AQUEOUS MIXTURE)	***S14***
ESI Section 7 EXTRACTION OF ACETONE FROM AQUEOUS STREAM INTO TOLUENE: MEASUREMENT	***S16***
ESI section 8 DEVELOPMENT OF ASPEN MODEL	***S18***
ESI Section 9 – SEQUENTIAL BATCH EXTRACTION PREDICTIONS	***S21***
ESI Section 10 – CHANGES IN VOLUME OF ORGANIC PHASE DURING EXTRACTION OF ACETONE FROM AQUEOUS TO TOLUENE PHASE	***S22***
**ESI REFERENCES**	***S23***

ESI Section 1 – VALVE AND PUMP SYSTEM

S1.1 Valve system

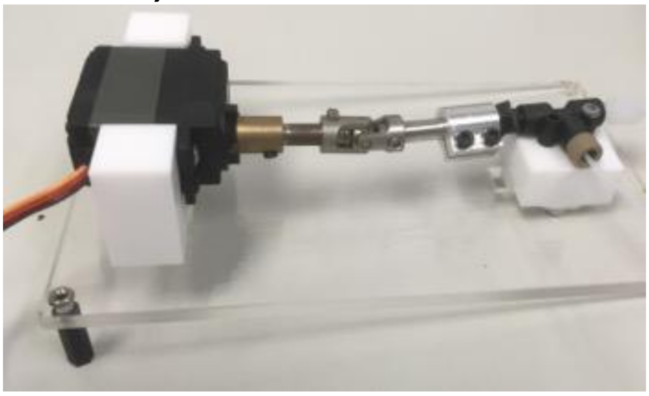

Figure S1 : Valve to servo connection

The needle valve is an Idex P-445 Micro-Metering Valve with ¼-28 UNF fittings. The valve connects to a servo motor via a universal joint. The servo motor was a Turnigy TGY-6114MD, specs of the motor are given in table S1. A back-pressure regulator (BPR) was attached to the opposite outlet. The BPR used was a P-791 (1.4 bar).

Table S1: Specifications of the Turnigy TGY-6114MD servo motor

**Turnigy TGY-6114MD Specifications:**	
Voltage range:	4.8-6 V
Speed:	0.6-0.72 sec/360°
Torque:	12.32-14.5 kg
Idle current:	0.12-0.14A
Working current:	0.4-0.5A
Stall current	1.8-2.2A
No turns	Up to 6

S1.2 Pump system

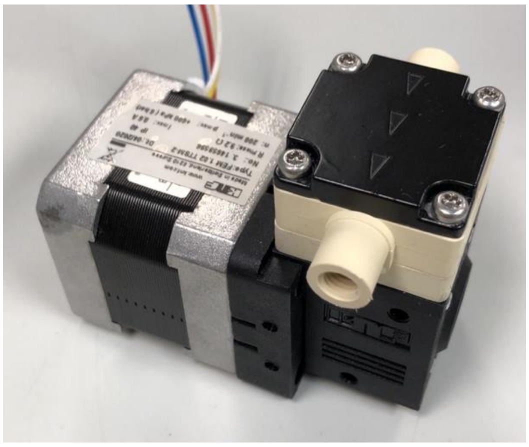

Figure S2: KNF FEM 1.02 pump used for outlet control system

The FEM 1.02 diaphragm pump is controlled by a stepper motor which was connected to an Arduino control circuit via a DRV8825 stepper motor driver and RAMPS 1.4 shield. The specifications of the pump are given in table S2. The electrical control circuitry of the valve system and pump system are described in sections S2.1 and S2.2.

Table S2: Specifications of the KNF FEM 1.02 pump

**KNF FEM 1.02 pump Specifications:**	
Pump head material:	PVDF
Diaphragm material:	PTFE
Valves material:	FFKM
Flow rate:	0.2 – 20 ml/min
Nominal stroke volume:	180 µl
Max pressure	6 Bar
Voltage	12 – 24 V
Load max	0.5 A

ESI Section 2 – ELECTRONIC CIRCUITS AND INTEFACE WITH FLUID CIRCUIT

S2.1: Valve system

The electronic housing and physical circuitry for the valve system (Figure S1) is shown in Figure S3. The system utilised an Arduino Uno Rev 3. The tentacle shield sits on top of the Arduino and uses its I2C capabilities to communicate with the EZO conductivity circuit. The tentacle shield has two BNC connectors which can connect to many standard conductivity probes or electrodes. In our setup we used a BNC cable attached to two steel electrodes of approximately 1 mm diameter which could be connected to the separator with a standard UNF1/4-28 HPLC connection. The Arduino was powered by via the USB port connected to a laptop. The servo was powered by an external power adapter 5 V, 2 A. Table S3 lists the main electrical components used in this setup.

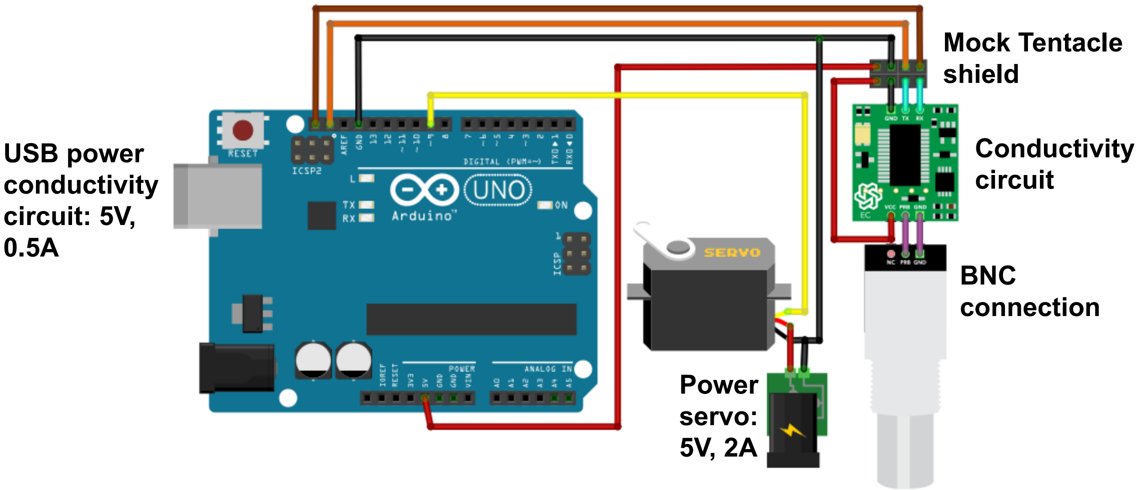

Figure S3: Simplified schematic of valve control system (produced using Fritzing software)

Table S3: Main electrical components of valve system

Components name	Description
Arduino Uno Rev 3	Open source microcontroller
Tentacle shield mini	Host and isolation for sensor circuits
EZO conductivity circuit	Conductivity sensor circuit
Turnigy TGY-6114MD servo motor	6 rotation servo motor

S2.2 Pump system

The electronic housing and physical circuitry for the pump system (Figure S2) used to control the separator outlet flow is shown in Figure S4. The system utilised an Arduino Mega 2560 Rev 3. The Ramps 1.4 stepper motor driver shield sits on top of the Arduino mega. The Ramps 1.4 connects to several of the Arduino pins which are then used to control the stepper motors which drive the diaphragm pumps. Multiple stepper motor drivers can be connected to the Ramps 1.4 board meaning up to five pumps can be connected to the system. The Arduino mega and stepper motors are powered by a 12 V, 5 A power adapter. The Ramps 1.4 board connects with the Arduino I2C circuitry and can therefore connect to the Tentacle shield which houses the conductivity circuits. Up to four conductivity circuits can be attached to the system, however this can be expanded if two or more tentacle shields are stacked. Table S4 details the main components of the system.

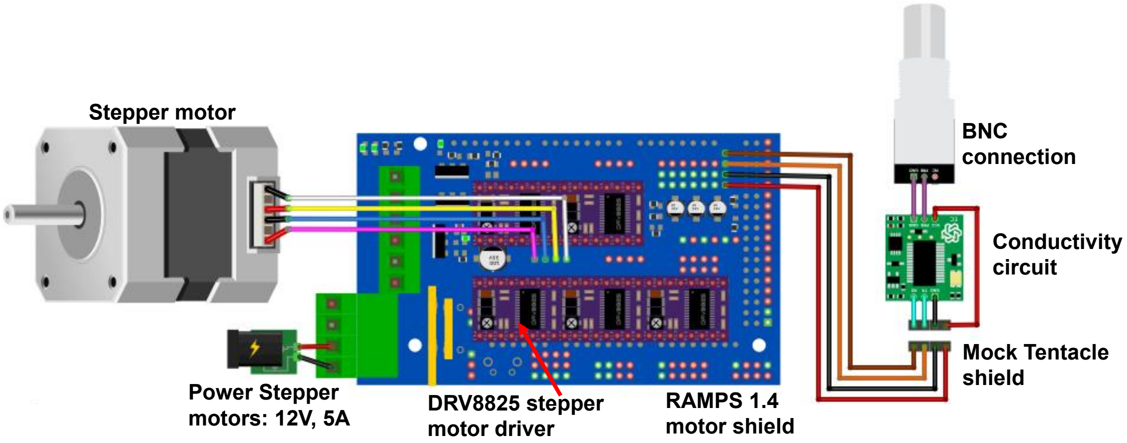

Figure S4: Simplified schematic of pump control system (produced using Fritzing software)

Table S4: Main electrical components of pump system

Components name	Description
Arduino Mega 2560 Rev 3	Open source microcontroller
Tentacle shield	Host and isolation for sensor circuits
EZO conductivity circuit	Conductivity sensor circuit
Ramps 1.4 motor shield	Motor shield for connecting multiple stepper motor drivers
DRV8825 stepper motor driver	Stepper motor driver for control of stepper motors
KNF FEM 1.02 diaphragm pump	Diaphragm pump – pump actuation via stepper motor

ESI Section 3 – CONTROL SYSTEM DEVELOPMENT

S3.1 Development of PID Control System

(i) Defining the operating setpoint:

To establish this setpoint automatically, an initialisation procedure was developed where the pump was stopped, or the valve fully closed, allowing the conductivity probe to become fully submersed in the denser aqueous phase giving a readout of the maximum conductivity of the aqueous solution. Figure S5, S6 shows the typical conductance measurements during this initialisation phase: at point A, the outlet aqueous flow is stopped, giving a rise in the conductance at time B to some maximum value C. The new conductance setpoint, D, was then set at 90% of this value.

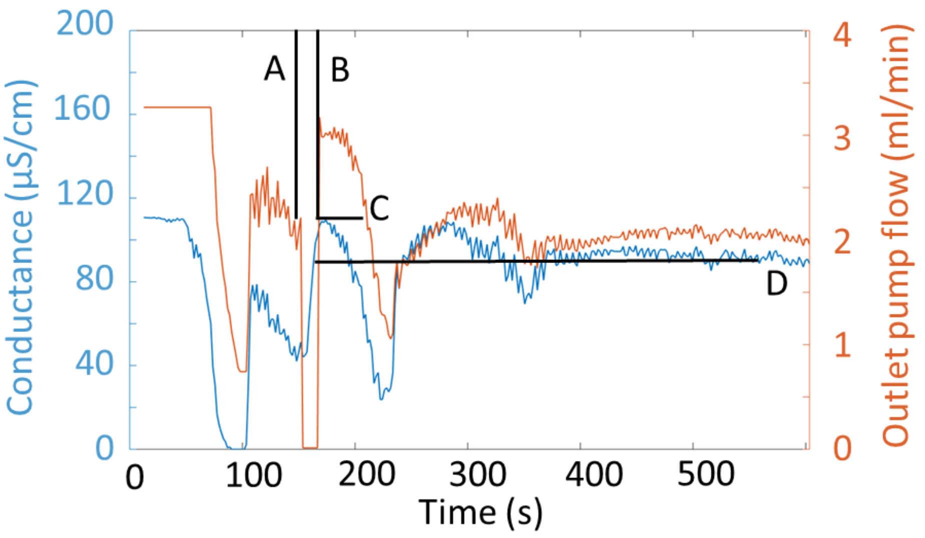

Figure S5: Conductance measurements and pump flow during automatic setpoint adjustment where aqueous flow is set to zero (A to B) to give maximum conductivity value (C) before an updated conductance is set (D).

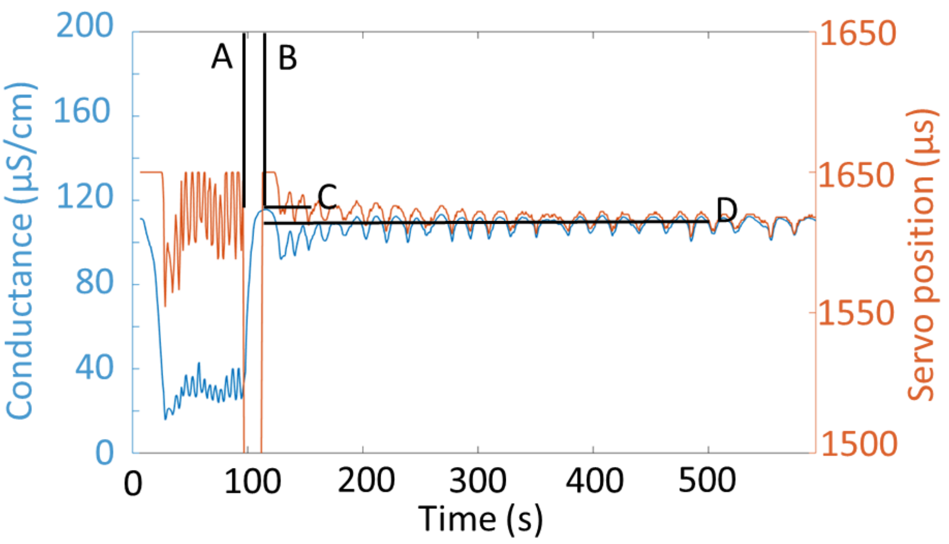

Figure S6: Conductance measurements and servo position during automatic setpoint adjustment where aqueous flow is set to zero (A to B) to give maximum conductivity value (C) before an updated conductance is set (D).

(ii) Identifying the PID constants: with the setpoint now established, the PID constants K_P, K_I and K_D were determined using the flow arrangement in Figure 3(a,b) (main paper), using the water-toluene system, with a flow rate of 4ml/min. With K_d = {0}, twelve experiments were run for all combinations of K_P={0.1,1,10} and K_I={0.01,0.1,1,10}, allowing the standard deviation of the pump flow or valve position and the conductivity measurement to be analysed. 200 data points (measured at a frequency of 1 Hz) were collected, starting 1 minute after the setpoint was found. The lowest standard deviation of the conductivity (corresponding to the most uniform value of the valve position or pump speed) was found with a K_P value of 1 and a K_I value of 0.05. .

The K_D constant was now introduced. A reduced window was considered, with all combinations of KP={0.5,1,2}, K_I={0.05} and K_D={0.1, 0.5,1}, with the final parameters of K_P = 1, K_I = 0.05 and K_D = 0.5 giving the lowest standard deviation of either pump speed or valve position indicating the most constant operating conditions. These parameters were found to work across all subsequent tests.

With K_d = {0}, twelve experiments were run for all combinations of K_P={0.1,1,10} and K_I={0.01,0.1,1,10}, allowing the standard deviation of the pump flow or valve position and the conductivity measurement to be analysed. A total of 200 data points measured at a frequency of 1 Hz were collected, starting one minute after the setpoint was found. The lowest standard deviation of the conductivity (corresponding to the most uniform value of the valve position or pump speed) was found with a K_P value of 1 and a K_I value of 0.05

The K_D constant was now introduced. A reduced window was considered, with all combinations of K_P={0.5,1,2}, K_I={0.05} and K_D={0.1, 0.5,1}, with the final parameters of K_P = 1, K_I = 0.05 and K_D = 0.5 giving the lowest standard deviation of either pump speed or valve position indicating the most constant operating conditions. These parameters were found to work across all subsequent tests.

S3.2 Standard Deviation of Conductivity and Valve Position

Figure S7 and S8 show example plots of how the standard deviation varies across the parameter space (P, I, D values varied). Low values of standard deviation give the most stable valve position (least wear) and most constant pump speed.

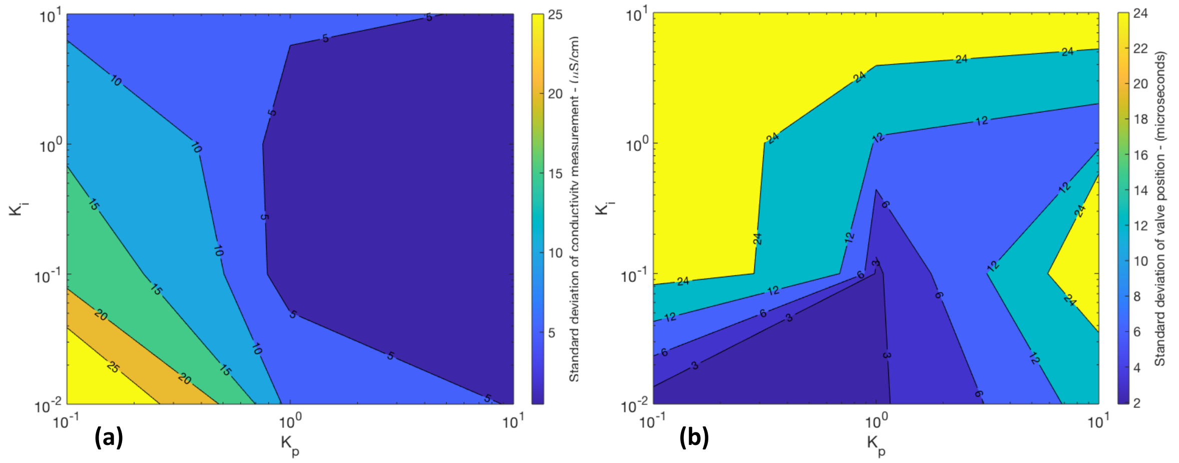

Figure S7: Standard deviation of (a) conductivity measurement and (b) valve position depending on K_P and K_i.

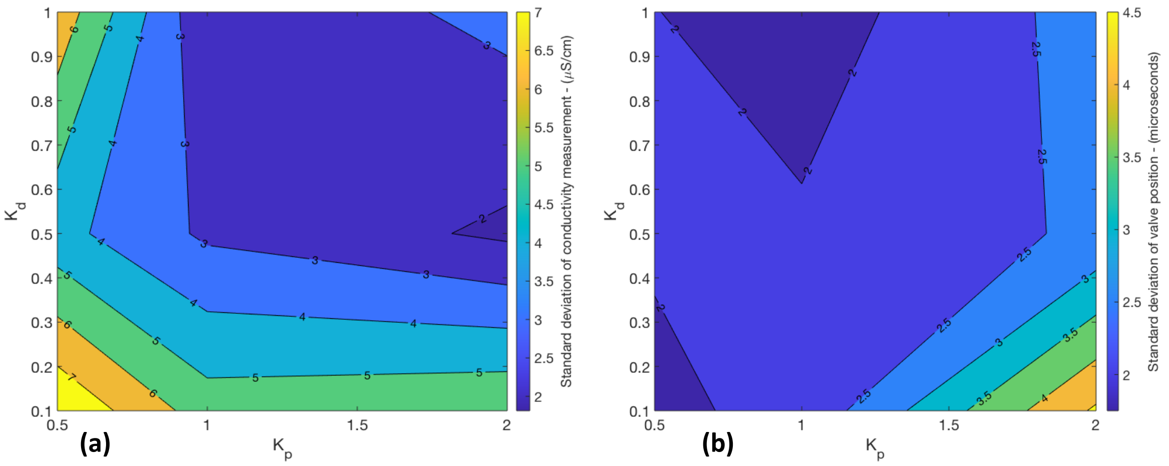

Figure S8: Standard deviation of (a) conductivity measurement and (b) valve position depending on K_P and K_d.

ESI Section 4 – MEASUREMENT PROTOCOL FOR FILTER MEDIA CHARACTERISTICS

Filter media characteristics:

The coalescing filter material used in this study was a meltblown PBT provided by Mogul Co. Ten samples were used to characterise the area density, thickness and permeability. Area density was measured according to BS EN 29073-1:1992 [S1] with ten 5x25 cm square samples, five of which were cut in the machine direction (MD) and 5 in the cross direction (CD). Material thickness was also measured according to BS EN 29073-2:1992 [S2] on these samples. The permeability, which is a measure of how easily a fluid can pass through a material was measured for ten 5 cm^2 samples with air as the fluid in accordance with BS EN ISO 9073-15:2008 [S3] using a FX 3300 LabAir IV; the intrinsic permeability was calculated from the air permeability data using equation (S4.1) where k (m^2) is the intrinsic permeability, U (m/s) the air velocity, μ (Pa.s) the viscosity of air, t (m) the thickness of the media t and ΔP (Pa) is the pressure drop across the fabric. With this method the pressure is fixed to 125 Pa and the resulting flow rate used to calculate U. The porosity Ø was calculated from the area density (GSM) and thickness data using equation (S4.2), with the density ρ of PBT of 1310 kg/m^3. The mean flow pore size, where half of the flow is through pores smaller than this (and half of the flow is through pores larger than this) was found using a POROLUX 100F and measured according to ASTM F316 – 03 (2011) [S4] over five samples.

$$k = \frac{U\mu t}{\Delta P} \tag{S4.1}$$

$$\emptyset = \left(1 - \frac{\rho t}{GSM}\right) \times 100 \tag{S4.2}$$

A Kruss droplet shape analyser was used to measure the advancing contact angle on the samples. The contact angle between a droplet of liquid and a surface is a measure of wettability. A large contact angle (greater than 90°) indicates low wetting of the surface and a small contact angle indicates high wetting of a surface. The protocols and best practices for contact angle analysis is set out by Huhtamäki et al (2018)[S5].

ESI Section 5 – SEPARATION DYNAMICS OF CLASS I SYSTEM (PURE ORGANIC / AQUEOUS MIXTURE)

The 12 ml samples collected from the coalescence filter and from the membrane separator were analysed using an imaging method. The number of pixels in the vertical direction of aqueous phase and organic phase were measured from the top of each sample to the bottom of each sample. In figure S9 and S10 the left sample is the organic outlet, and the right sample is the aqueous sample in each pair. The phase ratio was measured as organic/aqueous and the total flow rate is the sum of the aqueous and organic inlet flow rates.

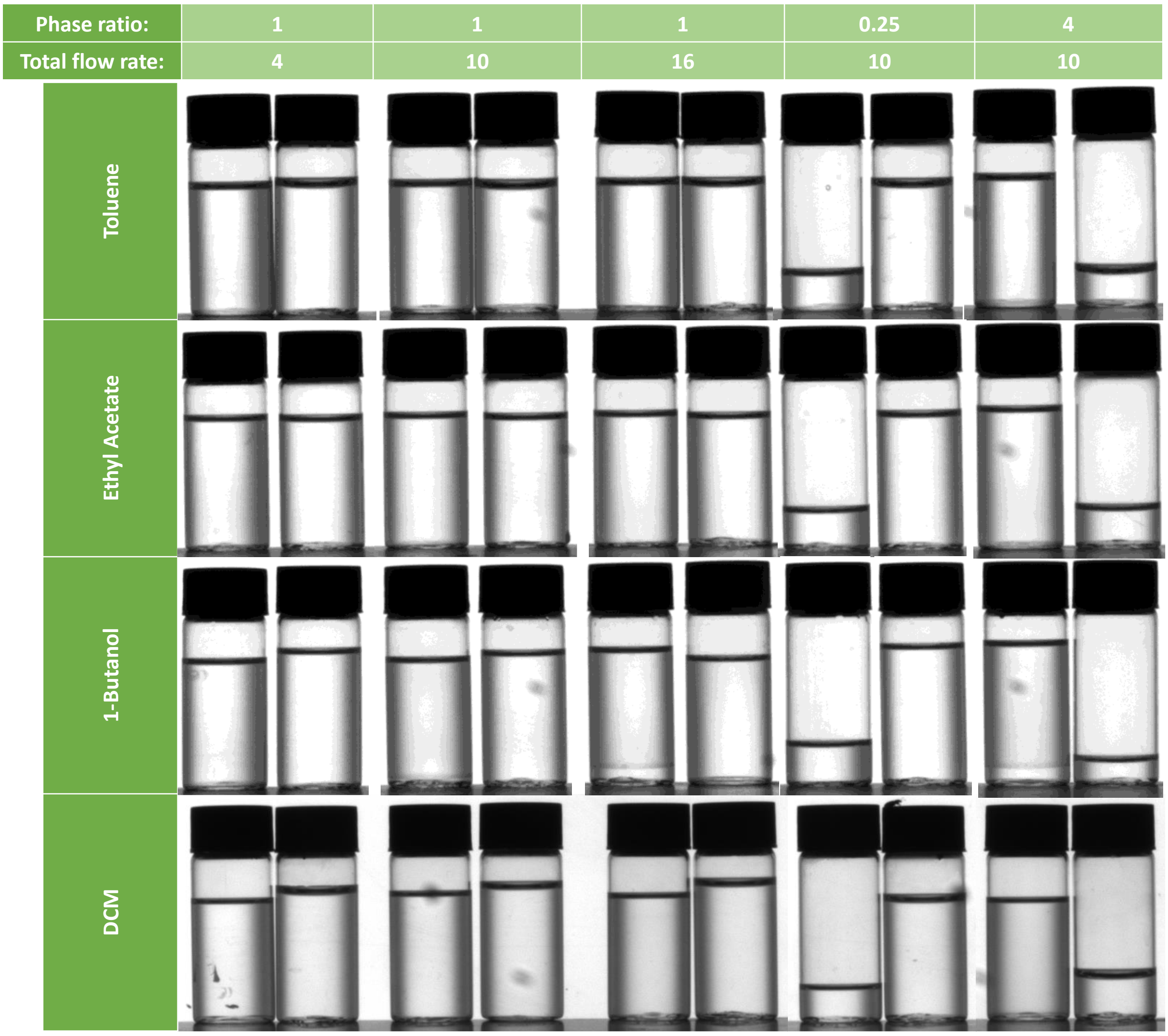

Figure S9: Images of samples taken from Organic and Aqueous outlets (left - organic, right – aqueous in each pair) of the coalescing filter

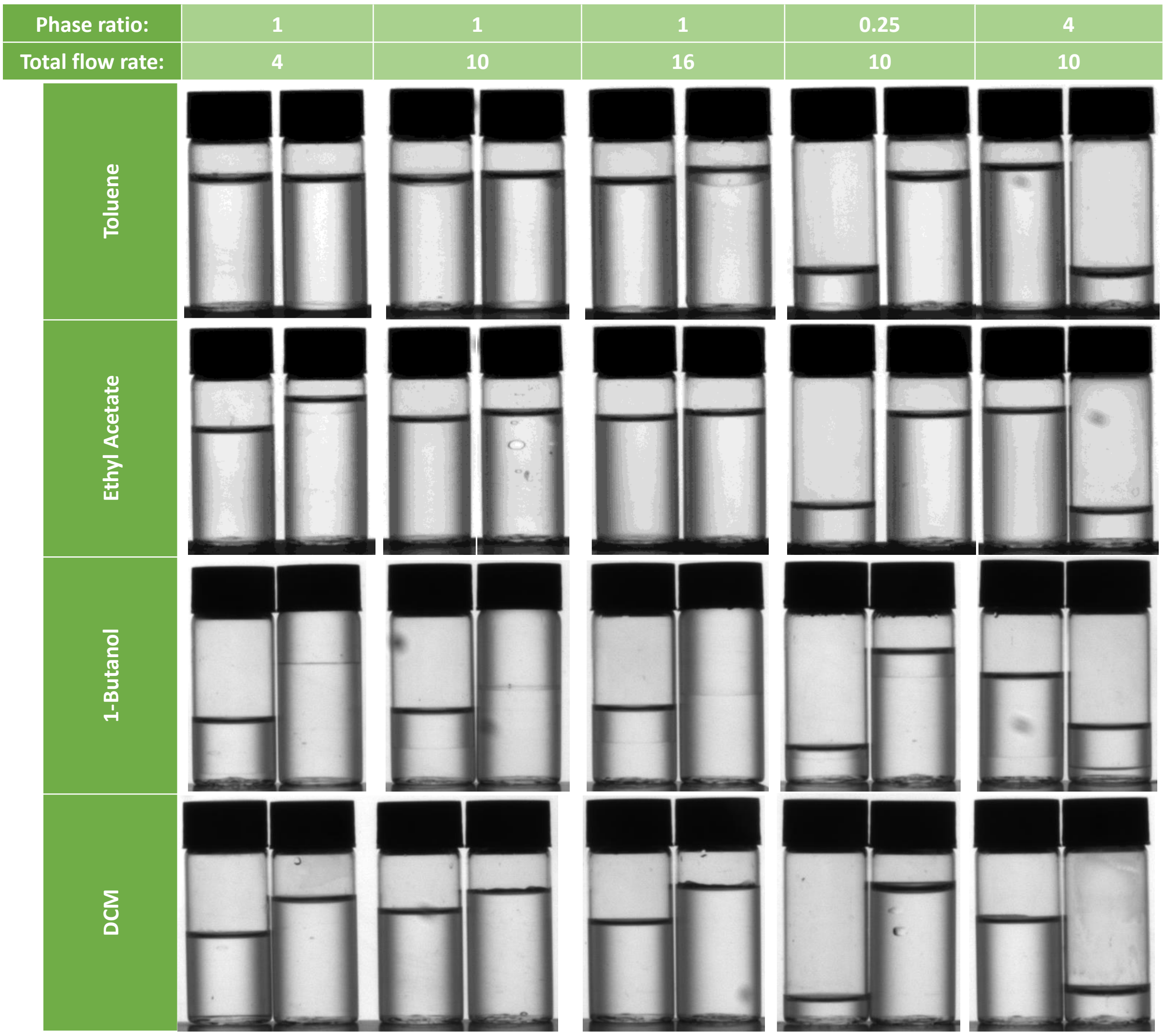

Figure S10: Images of samples taken from Organic and Aqueous outlets (left - organic, right – aqueous in each pair) of the membrane separator

Measured pixel lengths

The vertical pixel that corresponded to the top of the organic phase and the bottom of the organic phase in each sample and the top of the aqueous phase and bottom of the aqueous phase from each sample was recorded. The number of pixels between the top and bottom of each phase was calculated. The % of each phase in each outlet was then calculated from this. Tables S5-S8 present this data for the coalescing separator samples and tables S9-S12 present this data for the membrane separator samples.

Table S5: Pixel measurements of Toluene-water samples from coalescing filter.

	Top - Org	Bottom - Org	Pixel Length	Top - Aq	Bottom - Aq	Pixel Length	Vial total (pixels)	% Organic	% Water
**Toluene - 2 ml/min - F**	498	684	186	0	0	0	186	100	0
**Water - 2 ml/min - F**	0	0	0	496	682	186	186	0	100
**Toluene - 5 ml/min - F**	492	678	186	0	0	0	186	100	0
**Water - 5 ml/min - F**	0	0	0	498	678	180	180	0	100
**Toluene - 8 ml/min - F**	490	678	188	0	0	0	188	100	0
**Water - 8 ml/min - F**	0	0	0	494	676	182	182	0	100
**Toluene - 2 ml/min - PR = 0.25**	640	684	44	0	0	0	44	100	0
**Water - 8 ml/min - PR = 0.25**	0	0	0	500	682	182	182	0	100
**Toluene - 8 ml/min - PR = 4**	488	678	190	0	0	0	190	100	0
**Water - 2 ml/min - PR = 4**	0	0	0	638	676	38	38	0	100

Table S6: Pixel measurements of Ethyl acetate-water samples from coalescing filter.

	Top - Org	Bottom - Org	Pixel Length	Top - Aq	Bottom - Aq	Pixel Length	Vial total (pixels)	% Organic	% Water
**Ethyl Acetate - 2 ml/min - F**	492	686	194	0	0	0	194	100	0
**Water - 2 ml/min - F**	0	0	0	494	680	186	186	0	100
**Ethyl Acetate - 5 ml/min - F**	488	682	194	0	0	0	194	100	0
**Water - 5 ml/min - F**	0	0	0	490	680	190	190	0	100
**Ethyl Acetate - 8 ml/min - F**	486	680	194	0	0	0	194	100	0
**Water - 8 ml/min - F**	0	0	0	490	674	184	184	0	100
**Ethyl Acetate - 2 ml/min - PR = 0.25**	634	682	48	0	0	0	48	100	0
**Water - 8 ml/min - PR = 0.25**	0	0	0	488	680	192	192	0	100
**Ethyl Acetate - 8 ml/min - PR = 4**	480	678	198	0	0	0	198	100	0
**Water - 2 ml/min - PR = 4**	0	0	0	632	674	42	42	0	100

Table S7: Pixel measurements of 1-Butanol-water samples from coalescing filter.

	Top - Org	Bottom - Org	Pixel Length	Top - Aq	Bottom - Aq	Pixel Length	Vial total (pixels)	% Organic	% Water
**Butanol- 2 ml/min - TF**	502	686	184	0	0	0	184	100	0
**Water - 2 ml/min - TF**	0	0	0	486	684	198	198	0	100
**Butanol - 5 ml/min - TF**	498	680	182	0	0	0	182	100	0
**Water - 5 ml/min - TF**	0	0	0	488	680	192	192	0	100
**Butanol - 8 ml/min - TF**	484	660	176	660	680	20	196	89.7959184	10.2041
**Water - 8 ml/min - TF**	0	0	0	496	676	180	180	0	100
**Butanol - 2 ml/min - PR = 0.25**	636	686	50	0	0	0	50	100	0
**Water - 8 ml/min - PR = 0.25**	0	0	0	482	682	200	200	0	100
**Butanol - 8 ml/min - PR = 4**	474	660	186	660	680	20	206	90.2912621	9.70874
**Water - 2 ml/min - PR = 4**	0	0	0	654	680	26	26	0	100

Table S8: Pixel measurements of DCM-water samples from coalescing filter.

	Top - Org	Bottom - Org	Pixel Length	Top - Aq	Bottom - Aq	Pixel Length	Vial total (pixels)	% Organic	% Water
**DCM - 2 ml/min - NF**	430	594	164	0	0	0	164	100	0
**Water - 2 ml/min - NF**	0	0	0	420	592	172	172	0	100
**DCM - 5 ml/min - NF**	420	596	176	0	0	0	176	100	0
**Water - 5 ml/min - NF**	0	0	0	410	590	180	180	0	100
**DCM - 8 ml/min - NF**	424	588	164	0	0	0	164	100	0
**Water - 8 ml/min - NF**	0	0	0	408	586	178	178	0	100
**DCM - 2 ml/min - PR = 0.25**	552	594	42	0	0	0	42	100	0
**Water - 8 ml/min - PR = 0.25**	0	0	0	432	590	158	158	0	100
**DCM - 8 ml/min - PR = 4**	430	536	106	0	0	0	106	100	0
**Water - 2 ml/min - PR = 4**	0	0	0	536	590	54	54	0	100

Table S9: Pixel measurements of Toluene-water samples from membrane separator.

	Top - Org	Bottom - Org	Pixel Length	Top - Aq	Bottom - Aq	Pixel Length	Vial total (pixels)	% Organic	% Water
**Toluene - 2 ml/min - Phob**	540	724	184	0	0	0	184	100	0
**Water - 2 ml/min - Phob**	0	0	0	540	726	186	186	0	100
**Toluene - 5 ml/min - Phob**	538	722	184	0	0	0	184	100	0
**Water - 5 ml/min - Phob**	0	0	0	532	722	190	190	0	100
**Toluene - 8 ml/min - Phob**	544	728	184	0	0	0	184	100	0
**Water - 8 ml/min - Phob**	524	546	22	546	724	178	200	11	89
**Toluene - 2 ml/min - PR = 0.25 - phob**	688	732	44	0	0	0	44	100	0
**Water - 8 ml/min - PR = 0.25 - phob**	0	0	0	546	728	182	182	0	100
**Toluene - 8 ml/min - PR = 4 - phil**	522	726	204	0	0	0	204	100	0
**Water - 2 ml/min - PR = 4 - phil**	0	0	0	688	722	34	34	0	100

Table S10: Pixel measurements of Ethyl acetate-water samples from membrane separator.

	Top - Org	Bottom - Org	Pixel Length	Top - Aq	Bottom - Aq	Pixel Length	Vial total (pixels)	% Organic	% Water
**Ethyl Acetate - 2 ml/min - Phob**	560	726	166	0	0	0	166	100	0
**Water - 2 ml/min - Phob**	512	530	18	530	724	194	212	8.490566	91.5094
**Ethyl Acetate - 5 ml/min - Phob**	542	722	180	0	0	0	180	100	0
**Water - 5 ml/min - Phob**	0	0	0	532	718	186	186	0	100
**Ethyl Acetate - 8 ml/min - Phob**	540	720	180	0	0	0	180	100	0
**Water - 8 ml/min - Phob**	0	0	0	532	720	188	188	0	100
**Ethyl Acetate - 2 ml/min - PR = 0.25 - phob**	682	728	46	0	0	0	46	100	0
**Water - 8 ml/min - PR = 0.25 - phob**	0	0	0	540	726	186	186	0	100
**Ethyl Acetate - 8 ml/min - PR = 4 - phil**	530	726	196	0	0	0	196	100	0
**Water - 2 ml/min - PR = 4 - phil**	0	0	0	686	722	36	36	0	100

Table S11: Pixel measurements of 1-Butanol-water samples from membrane separator.

	Top - Org	Bottom - Org	Pixel Length	Top - Aq	Bottom - Aq	Pixel Length	Vial total (pixels)	% Organic	% Water
**Butanol - 2 ml/min - Phob**	530	600	70	600	610	10	80	87.5	12.5
**Water - 2 ml/min - Phob**	374	448	74	448	612	164	238	31.09244	68.9076
**Butanol - 5 ml/min - Phob**	516	566	50	566	608	42	92	54.34783	45.6522
**Water - 5 ml/min - Phob**	374	482	108	482	608	126	234	46.15385	53.8462
**Butanol - 8 ml/min - Phob**	512	558	46	558	608	50	96	47.91667	52.0833
**Water - 8 ml/min - Phob**	372	492	120	492	610	118	238	50.42017	49.5798
**Butanol - 2 ml/min - PR = 0.25 - phob**	570	584	14	584	610	26	40	35	65
**Water - 8 ml/min - PR = 0.25 - phob**	438	472	34	472	612	140	174	19.54023	80.4598
**Butanol - 8 ml/min - PR = 4 - phil**	470	578	108	578	610	32	140	77.14286	22.8571
**Water - 2 ml/min - PR = 4 - phil**	542	592	50	592	608	16	66	75.75758	24.2424

Table S12: Pixel measurements of DCM-water samples from membrane separator.

	Top - Org	Bottom - Org	Pixel Length	Top - Aq	Bottom - Aq	Pixel Length	Vial total (pixels)	% Organic	% Water
**DCM - 2 ml/min - Phob**	572	682	110	0	0	0	110	100	0
**Water - 2 ml/min - Phob**	0	0	0	524	681	157	157	0	100
**DCM - 5 ml/min - Phob**	540	680	140	0	0	0	140	100	0
**Water - 5 ml/min - Phob**	0	0	0	510	678	168	168	0	100
**DCM - 8 ml/min - Phob**	554	678	124	0	0	0	124	100	0
**Water - 8 ml/min - Phob**	0	0	0	506	678	172	172	0	100
**DCM - 2 ml/min - PR = 0.25 - phob**	654	680	26	0	0	0	26	100	0
**Water - 8 ml/min - PR = 0.25 - phob**	0	0	0	508	680	172	172	0	100
**DCM - 8 ml/min - PR = 4 - phil**	554	686	132	0	0	0	132	100	0
**Water - 2 ml/min - PR = 4 - phil**	0	0	0	652	684	32	32	0	100

ESI Section 6 – SEPARATION DYNAMICS OF CLASS II SYSTEM (SURFACTANT / ORGANIC / AQUEOUS MIXTURE)

The 10 ml samples collected from the coalescing separator during the emulsion separation tests have been presented in figure S11. There are three samples from each outlet (left three are organic and right three are aqueous, in each combination of HLD parameter and filter layers).

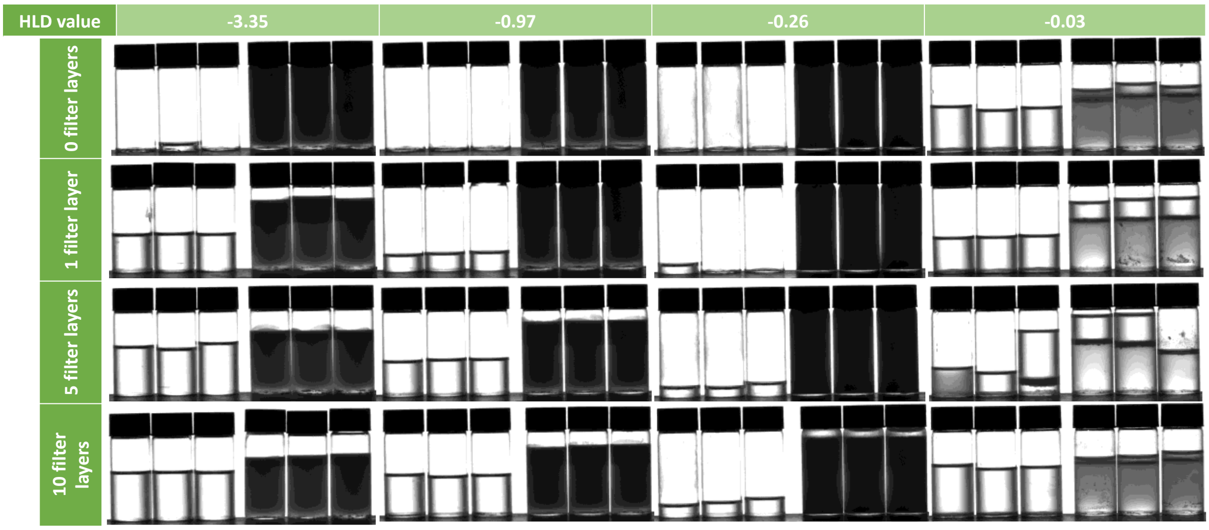

Figure S11: Images of samples taken from Organic and Aqueous outlets (left 3 - organic, right 3 – aqueous in each block) of the coalescing filter during the emulsion separation experiments.

The vertical pixel that corresponded to the top of the organic phase and the bottom of the organic phase and the top and bottom of the aqueous phase in the organic samples was recorded. The number of pixels between the top and bottom of each phase was calculated from this. The % of each phase in each outlet was then calculated from this using the knowledge that a 10 ml sample is 214 pixels long. Tables S13-S16 present this data for each HLD value and each different number of filter layers tested.

Table S13: Pixel measurements of Toluene-SDBS solution samples from coalescing filter outlets at HLD = -3.35.

HLD value	NacL concentration (M)	NaCl concentration (g/L)	Outlet	No Filter layers	Top - Org	Bottom - Org	Pixel Length	Top - Aq	Bottom - Aq	Pixel Length	Vial total (pixels)	% Organic	% Aqueous
-3.35	0.01	0.52	Organic	0	0	0	0	0	0	0	0	0	0
-3.35	0.01	0.52	Organic	0	818	832	14	0	0	0	14	7	0
-3.35	0.01	0.52	Organic	0	0	0	0	0	0	0	0	0	0
-3.35	0.01	0.52	Organic	1	700	832	132	0	0	0	132	62	0
-3.35	0.01	0.52	Organic	1	698	832	134	0	0	0	134	63	0
-3.35	0.01	0.52	Organic	1	700	830	130	0	0	0	130	61	0
-3.35	0.01	0.52	Organic	5	660	832	172	0	0	0	172	80	0
-3.35	0.01	0.52	Organic	5	668	830	162	0	0	0	162	76	0
-3.35	0.01	0.52	Organic	5	644	832	188	0	0	0	188	88	0
-3.35	0.01	0.52	Organic	10	662	834	172	0	0	0	172	80	0
-3.35	0.01	0.52	Organic	10	660	832	172	0	0	0	172	80	0
-3.35	0.01	0.52	Organic	10	660	830	170	0	0	0	170	79	0

Table S14: Pixel measurements of Toluene-SDBS solution samples from coalescing filter outlets at HLD = -0.97.

HLD value	NacL concentration (M)	NaCl concentration (g/L)	Outlet	No Filter layers	Top - Org	Bottom - Org	Pixel Length	Top - Aq	Bottom - Aq	Pixel Length	Vial total (pixels)	% Organic	% Aqueous
-0.97	0.18	10.50	Organic	0	0	0	0	0	0	0	0	0	0
-0.97	0.18	10.50	Organic	0	0	0	0	0	0	0	0	0	0
-0.97	0.18	10.50	Organic	0	0	0	0	0	0	0	0	0	0
-0.97	0.18	10.50	Organic	1	754	806	52	0	0	0	52	24	0
-0.97	0.18	10.50	Organic	1	746	804	58	0	0	0	58	27	0
-0.97	0.18	10.50	Organic	1	742	802	60	0	0	0	60	28	0
-0.97	0.18	10.50	Organic	5	682	802	120	0	0	0	120	56	0
-0.97	0.18	10.50	Organic	5	672	802	130	0	0	0	130	61	0
-0.97	0.18	10.50	Organic	5	670	800	130	0	0	0	130	61	0
-0.97	0.18	10.50	Organic	10	650	806	156	0	0	0	156	73	0
-0.97	0.18	10.50	Organic	10	656	798	142	0	0	0	142	66	0
-0.97	0.18	10.50	Organic	10	648	796	148	0	0	0	148	69	0

Table S15: Pixel measurements of Toluene-SDBS solution samples from coalescing filter outlets at HLD = -0.26.

HLD value	NacL concentration (M)	NaCl concentration (g/L)	Outlet	No Filter layers	Top - Org	Bottom - Org	Pixel Length	Top - Aq	Bottom - Aq	Pixel Length	Vial total (pixels)	% Organic	% Aqueous
-0.26	0.38	22.02	Organic	0	0	0	0	0	0	0	0	0	0
-0.26	0.38	22.02	Organic	0	0	0	0	0	0	0	0	0	0
-0.26	0.38	22.02	Organic	0	0	0	0	0	0	0	0	0	0
-0.26	0.38	22.02	Organic	1	0	0	0	0	0	0	0	0	0
-0.26	0.38	22.02	Organic	1	0	0	0	0	0	0	0	0	0
-0.26	0.38	22.02	Organic	1	766	790	24	0	0	0	24	11	0
-0.26	0.38	22.02	Organic	5	766	792	26	0	0	0	26	12	0
-0.26	0.38	22.02	Organic	5	768	788	20	0	0	0	20	9	0
-0.26	0.38	22.02	Organic	5	752	786	34	0	0	0	34	16	0
-0.26	0.38	22.02	Organic	10	760	790	30	0	0	0	30	14	0
-0.26	0.38	22.02	Organic	10	748	788	40	0	0	0	40	19	0
-0.26	0.38	22.02	Organic	10	734	784	50	0	0	0	50	23	0

Table S16: Pixel measurements of Toluene-SDBS solution samples from coalescing filter outlets at HLD = -0.03.

HLD value	NacL concentration (M)	NaCl concentration (g/L)	Outlet	No Filter layers	Top - Org	Bottom - Org	Pixel Length	Top - Aq	Bottom - Aq	Pixel Length	Vial total (pixels)	% Organic	% Aqueous
-0.03	0.47	27.68	Organic	0	636	786	150	0	0	0	150	70	0
-0.03	0.47	27.68	Organic	0	650	782	132	0	0	0	132	62	0
-0.03	0.47	27.68	Organic	0	640	782	142	0	0	0	142	66	0
-0.03	0.47	27.68	Organic	1	670	786	116	0	0	0	116	54	0
-0.03	0.47	27.68	Organic	1	668	782	114	0	0	0	114	53	0
-0.03	0.47	27.68	Organic	1	666	778	112	0	0	0	112	52	0
-0.03	0.47	27.68	Organic	5	698	780	82	780	788	8	90	38	4
-0.03	0.47	27.68	Organic	5	714	784	70	0	0	0	70	33	0
-0.03	0.47	27.68	Organic	5	556	724	168	724	784	60	228	79	28
-0.03	0.47	27.68	Organic	10	610	784	174	0	0	0	174	81	0
-0.03	0.47	27.68	Organic	10	622	782	160	0	0	0	160	75	0
-0.03	0.47	27.68	Organic	10	616	780	164	0	0	0	164	77	0

ESI Section 7 EXTRACTION OF ACETONE FROM AQUEOUS STREAM INTO TOLUENE: MEASUREMENT

At each flow rate combination, the system was left to run for 30 minutes before a sample of the organic outlet (extract) was taken to allow the system to reach steady state. In order to determine the organic outlet flow rate, an 8 ml sample was collected and the time required to collect this sample was recorded. A separate 5 ml sample of the organic outlet (extract) was collected for analysis using gas chromatography (GC).

To measure the amount of acetone extracted into toluene a GC method was created. This required a calibration curve in order to determine what peak area corresponded to what concentration of acetone. Therefore, a range of solutions with different concentrations of acetone and toluene were produced. Each sample was 10 ml in volume and had a different ratio of toluene to acetone. The weight and volume of acetone and toluene in each calibration sample has been presented in table S17 along with the peak area of each given by the GC method and the concentration of acetone in mg/ml calculated from the weight measurements of each solution. Figure S12(a) plots acetone concentration against peak area in order to determine the straight line equation that describes the relationship between them and figure S12(b) does the same but for the toluene concentration. The sample with concentration 39.43 mg/ml was omitted from the calibration curve because it was at a low concentration, much less than what was found during the actual extractions and it produced a large lever arm that influenced the straight lines angle (m in the straight-line equation y = mx + c).

Table S17: Weight, volume and concentration of calibration samples and their corresponding peak areas using GC

Toluene weight (mg)	Acetone weight (mg)	Toluene volume (ml)	Acetone volume (ml)	Concentration of acetone (mg/ml)	Toluene peak area	Acetone peak area
8235	8234	9.5	0.5	39.4	1348347	20092
7802	7800	9	1	78.6	1253709	40211
6938	6935	8	2.02	157.9	1096463	82086
6069	6065	7	3	235.4	966134	127331
5204	5199	6	4	313.5	816104	172058
4336	4330	5	5.01	392.2	693249	227873

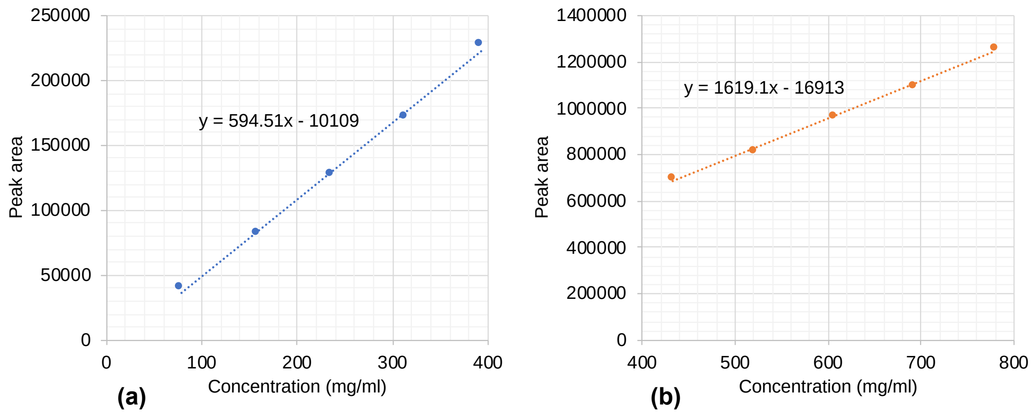

Figure S12: Calibration curve for (a) acetone concentration in acetone + toluene solution (omitting 39.42 mg/ml sample) and (b) toluene concentration in acetone + toluene solution (omitting the same sample).

The batch results were calculated under the assumption that toluene and water were immiscible, therefore all of the toluene would be present in the organic phase. Based on this assumption the volume of acetone in the organic phase after mixing with the aqueous could be calculated. Firstly the

concentration of toluene was found using the toluene calibration curve and peak areas. As the mass of toluene added to the solution was known for each phase ratio, dividing this value by the concentration would yield the total volume of the organic sample after mixing. Taking this value and multiplying it by the concentration of acetone in the organic phase (found via the acetone calibration curve) gave the weight of acetone in the organic phase. To obtain the percentage of acetone extracted, this weight was divided by the weight of acetone in the aqueous phase before mixing and multiplied by 100. Equation S7.1 describes this procedure.

$$\%\ \text{Acetone extracted} = \frac{C_{\text{Acetone,Ex}} \times \text{Weight}_{\text{Toluene,S}}}{C_{\text{Toluene,Ex}} \times \text{Weight}_{\text{Acetone,F}}} \times 100 \qquad \text{(S7.1)}$$

$C_{\text{Acetone,Ex}} =$	Concentration of acetone in the extract phase	(mg/ml)
$C_{\text{Toluene,Ex}} =$	Concentration of toluene in the extract phase	(mg/ml)
$\text{Weight}_{\text{Toluene,S}} =$	Weight of toluene (solvent phase)	(mg)
$\text{Weight}_{\text{Acetone,F}} =$	Weight of acetone (feed)	(mg)

To calculate the % of acetone extracted in the counter-current flow experiments equation S7.2 was used. The only difference was that concentration was calculated in mg/ml instead of kg/kg and flow rate in ml/min rather than L/min. The organic outlet (extract) flow rate was calculated from the time it took to fill an 8 ml volume and the aqueous feed flow rate was specified by the inlet aqueous pump speed.

$$\%\ \text{acetone extracted} = \frac{C_{\text{Acetone,Ex}} \times Q_{\text{Ex}}}{C_{\text{Acetone,F}} \times Q_{\text{F}}} \times 100 \qquad \text{(S7.2)}$$

$C_{\text{Acetone,Ex}} =$	Concentration of acetone in extract stream	(kg/kg)
$C_{\text{Acetone,F}} =$	Concentration of acetone in feed stream	(kg/kg)
$Q_{\text{Ex}} =$	Flow rate of extract stream	(L/min)
$Q_{\text{F}} =$	Flow rate of feed stream	(L/min)

ESI section 8 DEVELOPMENT OF ASPEN MODEL

Modelling method

Selection of a thermodynamic model suitable to the system in question is important for obtaining an accurate representation of that system. Al-Malah, 2017[S7] recommends the use of an activity coefficient model as they tend to perform well for systems with polar compounds at low pressures away from the critical region. Three such models were used to create a ternary phase diagram of toluene-acetone-water. These phase diagrams were compared to the extraction results obtained experimentally in batch to find the model that represented the real system most closely. The phase diagram that matched the batch results most closely was used in the subsequent process simulations for counter-current extraction.
The three thermodynamic models tested were:

- NRTL-RK (Non-Random Two Liquid with Redlich-Kwong equations)
- UNIFAC (Universal functional group activity coefficients)
- UNIQUAC (Universal Quasi-chemical model)

The three system components were set as water, acetone and toluene. When producing the ternary phase diagram the temperature was set at 25 ºC and a pressure of 1 bar. It was found that the UNIFAC model fit the batch experimental data best and so this method was used for the counter-current simulation. The ternary phase diagram produced by Aspen Plus V10 is given in figure S13.

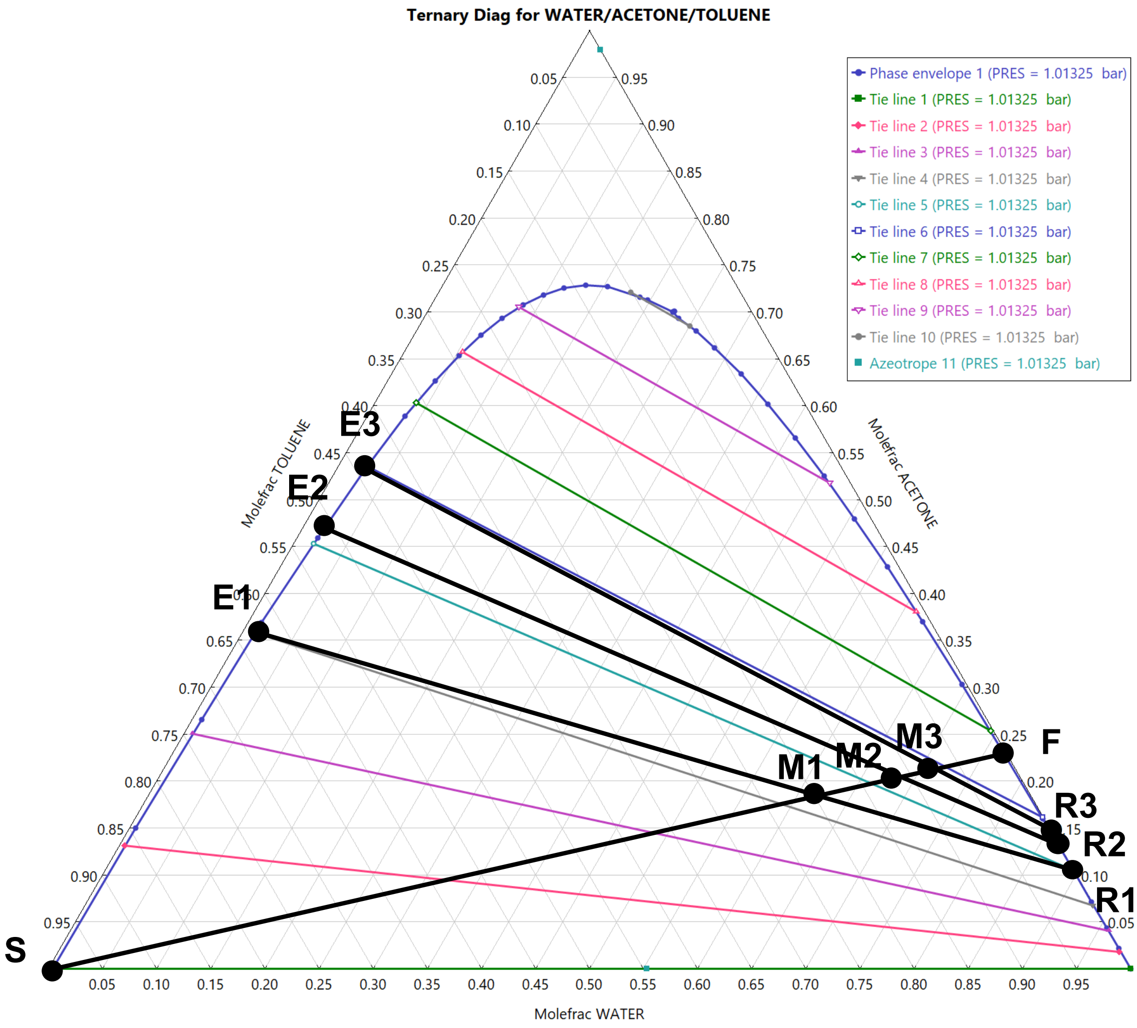

Figure S13: Ternary phase diagram of toluene-acetone-water system with overlayed extract and raffinate mole fractions at three phase ratios and the mixing point of each system.

The results from the batch extraction are overlayed on the ternary diagram so that the extract and raffinate found via experiments can be compared to the tie lines on the ternary diagram. The closer the direction of the tie lines matched with the extraction results, the better the model was deemed to be. In figure S13, S = Solvent composition, F = Feed composition, M_1, M_2, and M_3 are the mixing points found at phase ratios of 1, 2 and 3. E_1, E_2 and E_3 and R_1, R_2 and R_3 are the extract phase and raffinate phase compositions when the solvent and feed were mixed at phase ratios of 1, 2 or 3. As can be seen from figure S13 the tie lines produced by the UNIFAC model match closely the experimental data found at phase ratios 2 and 3 well. However, the model data deviates from the experimental data at a phase ratio of 1 as the raffinate phase had a higher concentration of acetone than predicted by the model. Out of the three models tested, this model fit the experimental data best and was therefore used for the counter-current extraction simulation.

The simulation model was tested at a phase ratio of 1, 2 and 3 (Aq/Org). The simulation was also set up as either a single stage extraction, 2 or 3 stages in counter-current. Figure S14 shows the 3 different flow configurations and Table S18 summarises the inlet stream compositions.

(a)

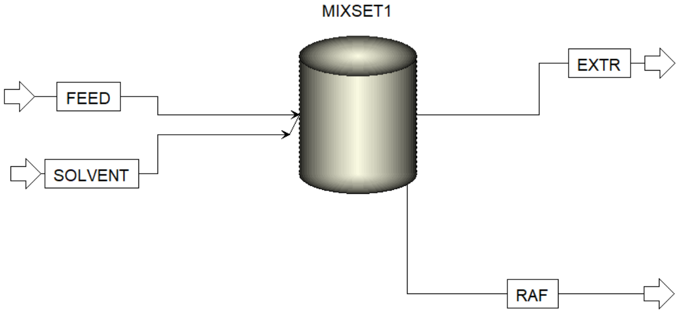

(b)

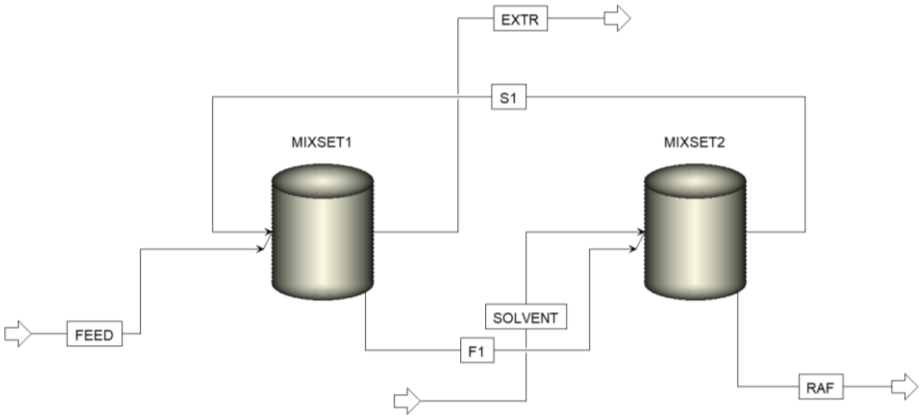

(c)

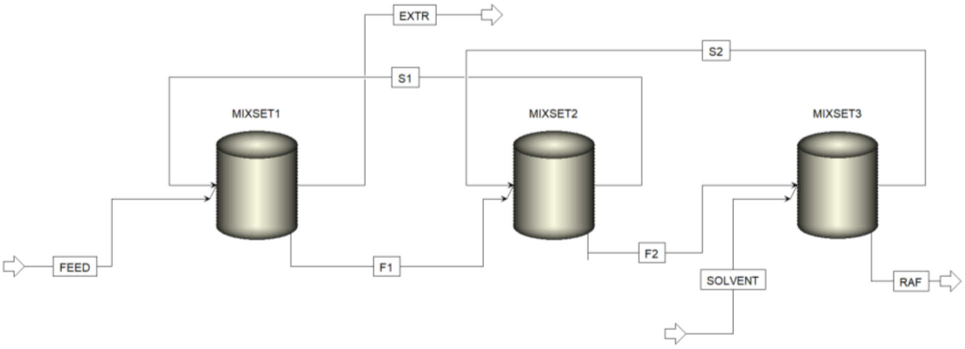

Figure S14: Counter-current flow simulation as (a) single stage, (b) two stage and (c) three stage extraction.

Table S18: Inlet flow compositions for each phase ratio and number of stages.

No of stages	Phase ratio (Aq/Org)	Feed flow rate (L/min)	Solvent flow rate (L/min)	Feed composition (kg/kg)	
1	1	1.5	1.5	Water	0.5
1	2	2	1	Acetone	0.5
1	3	2.25	0.75	**Solvent composition (kg/kg)**	
2	1	1.5	1.5		
2	2	2	1	Toluene	1
2	3	2.25	0.75		
3	1	1.5	1.5		
3	2	2	1		
3	3	2.25	0.75		

The outlet streams ('extr' and 'raf') composition was collected from the model output data. The weight fraction of the extract and feed streams combined with the stream flow rates gave the % of acetone extracted from the feed stream using equation S8.4.

$$\%\ \text{acetone extracted} = \frac{C_{\text{Acetone,Ex}} \times Q_{\text{Ex}}}{C_{\text{Acetone,F}} \times Q_{\text{F}}} \times 100 \qquad \text{(S8.4)}$$

$C_{\text{Acetone,Ex}} =$ Concentration of acetone in extract stream (kg/kg)
$C_{\text{Acetone,F}} =$ Concentration of acetone in feed stream (kg/kg)
$Q_{\text{Ex}} =$ Flow rate of extract stream (L/min)
$Q_{\text{F}} =$ Flow rate of feed stream (L/min)

ESI Section 9 – SEQUENTIAL BATCH EXTRACTION PREDICTIONS

Aqueous feed composition: water: acetone (kg/kg): 1:1
Toluene feed composition: toluene (kg/kg): 1

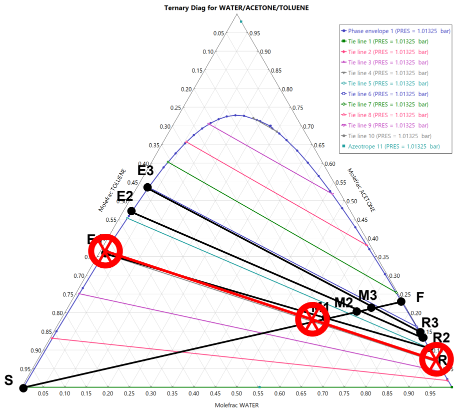

Single stage: Overall phase ratio (aqueous : toluene) 1:1

Stage 1: Aqueous: toluene 1:1

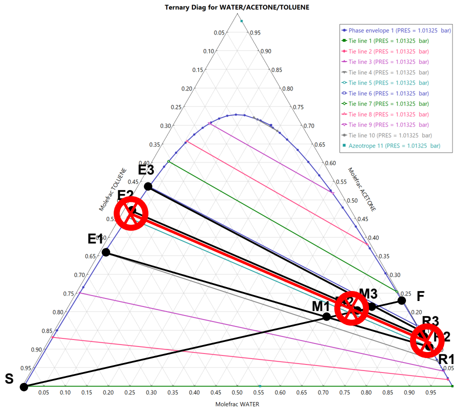

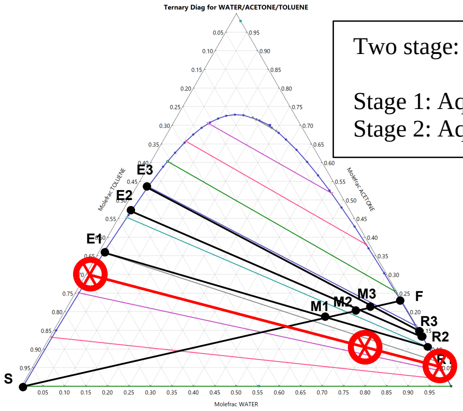

Two stage: Overall phase ratio (aqueous : toluene) 1:1

Stage 1: Aqueous: toluene 1:0.5
Stage 2: Aqueous (from stage 1): toluene 1: 0.5

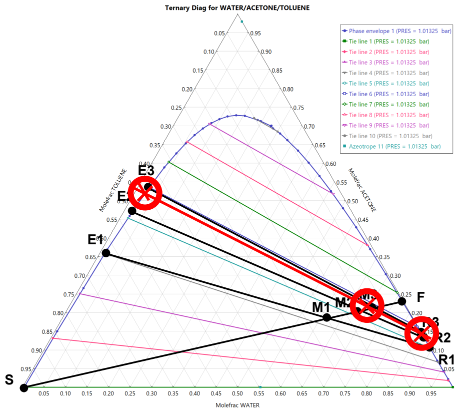

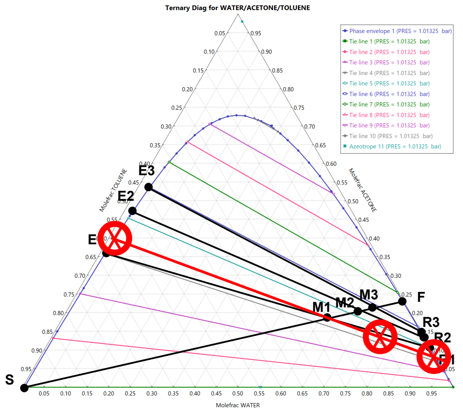

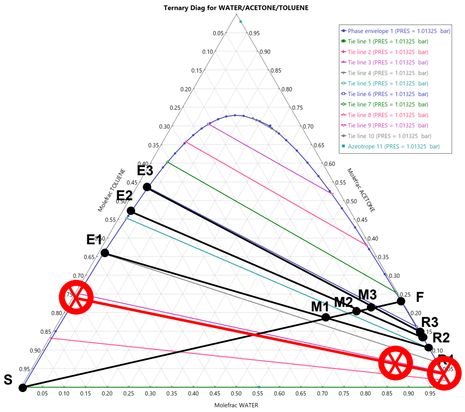

Three stage: Overall phase ratio (aqueous : toluene) 1:1

Stage 1: Aqueous: toluene 1:0.33
Stage 2: Aqueous (from stage 1): toluene 1:0.33
Stage 3: Aqueous (from stage 2): toluene 1:0.33

Figure S15: Tie diagrams for calculating sequential batch extraction compositions.

ESI Section 10 – CHANGES IN VOLUME OF ORGANIC PHASE DURING EXTRACTION OF ACETONE FROM AQUEOUS TO TOLUENE PHASE

Table S19: Inlet and outlet flow rates of the continuous extraction system and the % increase in organic phase flow rate determined from the experiments.

No of stages	Phase ratio (Aq/Org)	Feed flow rate (ml/min)	Solvent flow rate (ml/min)	Extract flow rate (ml/min)	% Organic flow rate increase
1	1	1.5	1.5	1.79	19
1	2	2	1	1.41	41
1	3	2.25	0.75	1.17	56
2	1	1.5	1.5	2.05	37
2	2	2	1	1.50	50
2	3	2.25	0.75	1.32	76
3	1	1.5	1.5	2.40	60
3	2	2	1	2.11	111
3	3	2.25	0.75	1.94	158

ESI REFERENCES

[S1] BS EN 29073-1:1992, ISO 9073-1:1989: Methods of test for nonwovens. Methods of test for nonwovens. Determination of mass per unit area. British Standards Institute: 1990.

[S2]. BS EN 29073-2:1992, ISO 9073-2:1989: Methods of test for nonwovens. Methods of test for nonwovens. Determination of thickness. British Standards Institute: 1990.

[S3]. BS EN ISO 9073-15:2008: Textiles. Test methods for nonwovens. Determination of air permeability. British Standards Institute: 2008.

[S4]. International, A., ASTM F316-03(2019), Standard Test Methods for Pore Size Characteristics of Membrane Filters by Bubble Point and Mean Flow Pore Test. West Conshohocken, PA, 2011.

[S5] Huhtamäki T, Tian X, Korhonen JT, Ras RHA. Surface-wetting characterization using contact-angle measurements. *Nature Protocols*. 2018/07/01 2018;13(7):1521-1538. doi:10.1038/s41596-018-0003-z

[S6] André BDH, Bosch H. *Industrial Separation Processes: Fundamentals*. Walter De Gruyter; 2013.

[S7] Al-Malah KIM. *Aspen plus : chemical engineering applications*. Wiley; 2017.
